# Systematic and computational identification of *Androctonus crassicauda* long non-coding RNAs

**DOI:** 10.1038/s41598-021-83815-8

**Published:** 2021-02-25

**Authors:** Fatemeh Salabi, Hedieh Jafari, Shahrokh Navidpour, Ayeh Sadat Sadr

**Affiliations:** 1 Department of Venomous Animals and Anti-Venom Production, Razi Vaccine and Serum Research Institute, Agricultural Research, Education and Extension Organization (AREEO), Ahvaz, Iran; 2 Department of Venomous Animals and Anti-Venom Production, Razi Vaccine and Serum Research Institute, Agricultural Research, Education and Extension Organization (AREEO), Karaj, Iran; 3 Aquaculture Research Center-South of Iran, Iranian Fisheries Science Research Institute, Agricultural Research, Education and Extension Organization (AREEO), Ahvaz, Iran

**Keywords:** Biotechnology, Computational biology and bioinformatics, Genetics, Zoology

## Abstract

The potential function of long non-coding RNAs in regulating neighbor protein-coding genes has attracted scientists’ attention. Despite the important role of lncRNAs in biological processes, a limited number of studies focus on non-model animal lncRNAs. In this study, we used a stringent step-by-step filtering pipeline and machine learning-based tools to identify the specific *Androctonus crassicauda* lncRNAs and analyze the features of predicted scorpion lncRNAs. 13,401 lncRNAs were detected using pipeline in *A. crassicauda* transcriptome. The blast results indicated that the majority of these lncRNAs sequences (12,642) have no identifiable orthologs even in closely related species and those considered as novel lncRNAs. Compared to lncRNA prediction tools indicated that our pipeline is a helpful approach to distinguish protein-coding and non-coding transcripts from RNA sequencing data of species without reference genomes. Moreover, analyzing lncRNA characteristics in *A. crassicauda* uncovered that lower protein-coding potential, lower GC content, shorter transcript length, and less number of isoform per gene are outstanding features of *A. crassicauda* lncRNAs transcripts.

## Introduction

The number of non-coding RNAs (ncRNAs) has significantly increased in recent years due to rapid development of RNA Sequencing (RNA-Seq), databases such as GENCODE, NONCODE, and RNAcenrtal and bioinformatics algorithms-free tools^1^. Many types of ncRNAs grouped due to their function, localization, and length, including transfer RNAs (tRNA), transfer-messenger RNA (tmRNA), ribosomal RNA (rRNA), small nucleolar RNAs (snoRNAs), small nuclear RNAs (snRNAs), small interfering RNAs (siRNAs), PIWI-interacting RNAs (piRNAs), promoter-associated RNAs (pRNAs), microRNAs (miRNAs), long non-coding RNAs (lncRNAs), circular RNAs (circRNA), signal recognition particle RNAs (SRP RNA), etc^2,3^. LncRNAs are a new and critical class of ncRNAs with a series of unique features. Compared to protein-coding mRNAs, a majority of lncRNAs have a shorter transcript size and lower GC content. Furthermore, LncRNAs are generally functional molecules transcribed from invertebrate to mammalian genomes but lack protein-coding ability^4–6^.

Currently, due to lncRNAs functionality in regulating neighbor protein-coding genes expression, mRNAs stability, post-translational modifications, translation, epigenetic modifications, DNA methylation, and their ability to interact with diverse macro-molecules^3, 5, 7^, they attracted the attention of scientists. Despite the important role of lncRNAs in biological processes, a limited number of studies focus on non-model animal lncRNAs. Various studies about insects investigated the transcriptome in the last decade, with lessened attention to lncRNAs^8–11^. Using deep RNA-seq technology, many lncRNAs were identified in several insects including 8096 putative lncRNAs in *Plutella xylostella*^4^, 11,810 lncRNAs in *Anopheles gambiae*^12^, 2949 lncRNAs in *Gambiae complex*^12^, 4689 novel lncRNA transcripts in *Ae. aegypti* and 6863 novel lncRNAs in the honey bee^13^. Roughly forty-three thousand known lncRNAs of the fruit fly have been registered in the NONCODEv4 database but have yet to be identified in other insects. Beyond those mentioned, more knowledge about scorpion venom compositions were achieved by high-throughput transcriptomic analyses of scorpion venom glands^9,14^; however, regulatory RNAs of venom gland biological processes have remained unknown. The employment of lncRNAs identification in scorpion adds new insights about the biological processes of the venom gland and facilitates the identification of regulatory factors. Nevertheless, no studies were conducted to predict the scorpion lncRNAs to date; therefore, to overcome these limitations, we used RNA sequencing (RNA-seq) to de novo assembled the scorpion transcriptome following by discovered specific *Androctonus crassicauda* lncRNAs using a stringent step-by-step filtering pipeline due to the main route of pipelines modeled for other species.

Moreover, we employed machine learning classifiers and alignment-free software not only to obtain high-confidence predictions of lncRNAs/mRNAs but also to validate our pipeline. For this purpose, several lncRNA prediction software were tested on the scorpion and fruit fly data sets to distinguish lncRNAs from protein-coding RNAs, including Coding Potential Calculator 2 (CPC2)^15^, Coding-Non-Coding Identifying Tool (CNIT)^16^, and a predictor of long non-coding RNAs and messenger RNAs due to improved k-mer scheme (PLEK)^17^. In general, we provide a powerful pipeline to predict lncRNAs in the scorpion and closely related species and describe the best lncRNA prediction tool tested on the scorpion dataset. Besides, our filtering pipeline combined with machine learning-based tools, helps researchers focus their efforts on highly validated known and novel lncRNAs in the scorpions. This study is the first comprehensive analysis and characterization of lncRNAs in the scorpions.

## Results

To predict lncRNAs in *A. crassicauda*, we collected samples from six male and female scorpions of varying age categories (mature and immature), and for identifying high confident lncRNA, generated paired-end RNA-seq libraries were analyzed. The sequence quality assay of male/female data of mature/immature scorpions is reported in supplementary figures F1–F6. 472 million clean reads were assembled into 952,725 transcripts (585,177 genes) by Trinity tool using default parameters^18,19^.

### Development of pipeline for identification of lncRNAs in scorpion transcriptome

**Figure 1:**
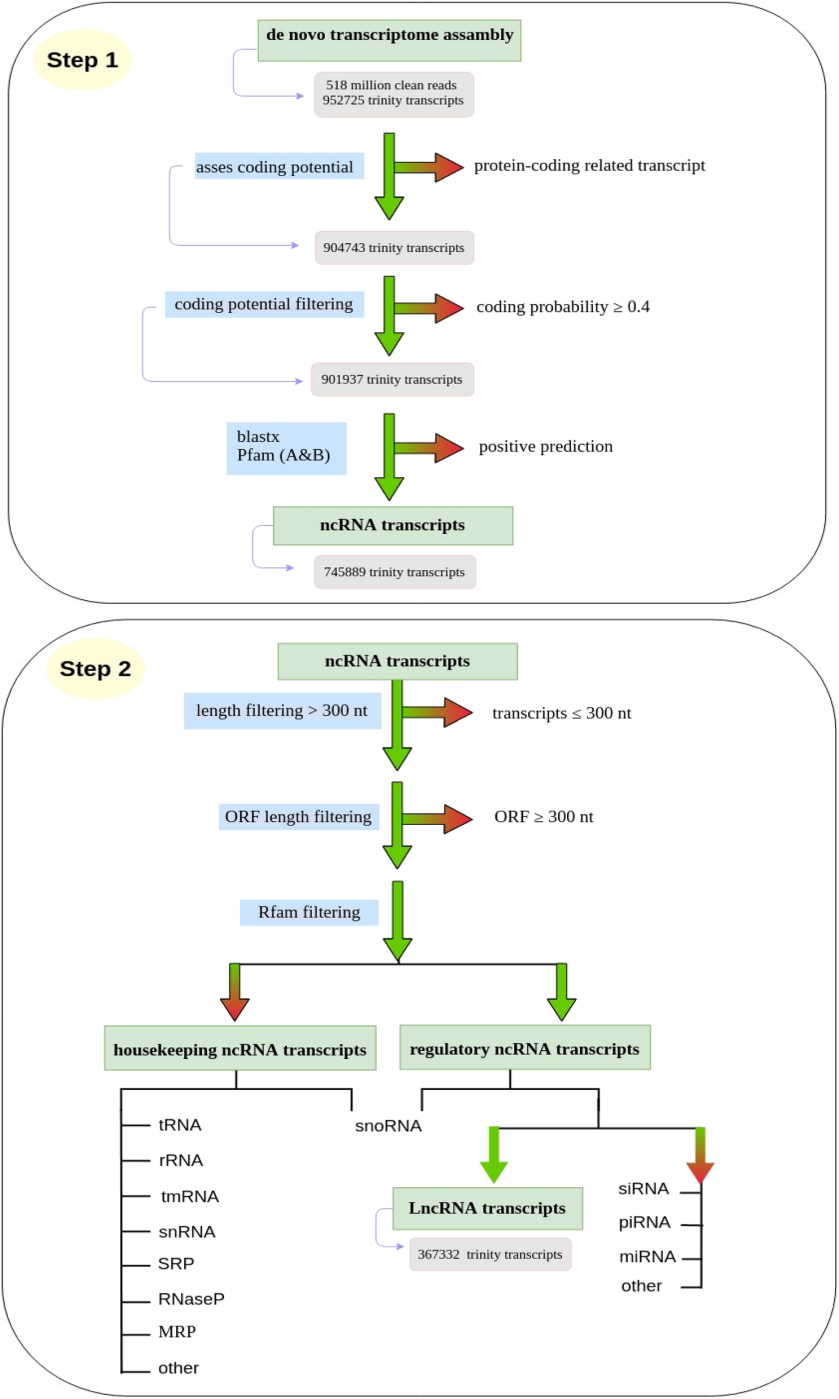

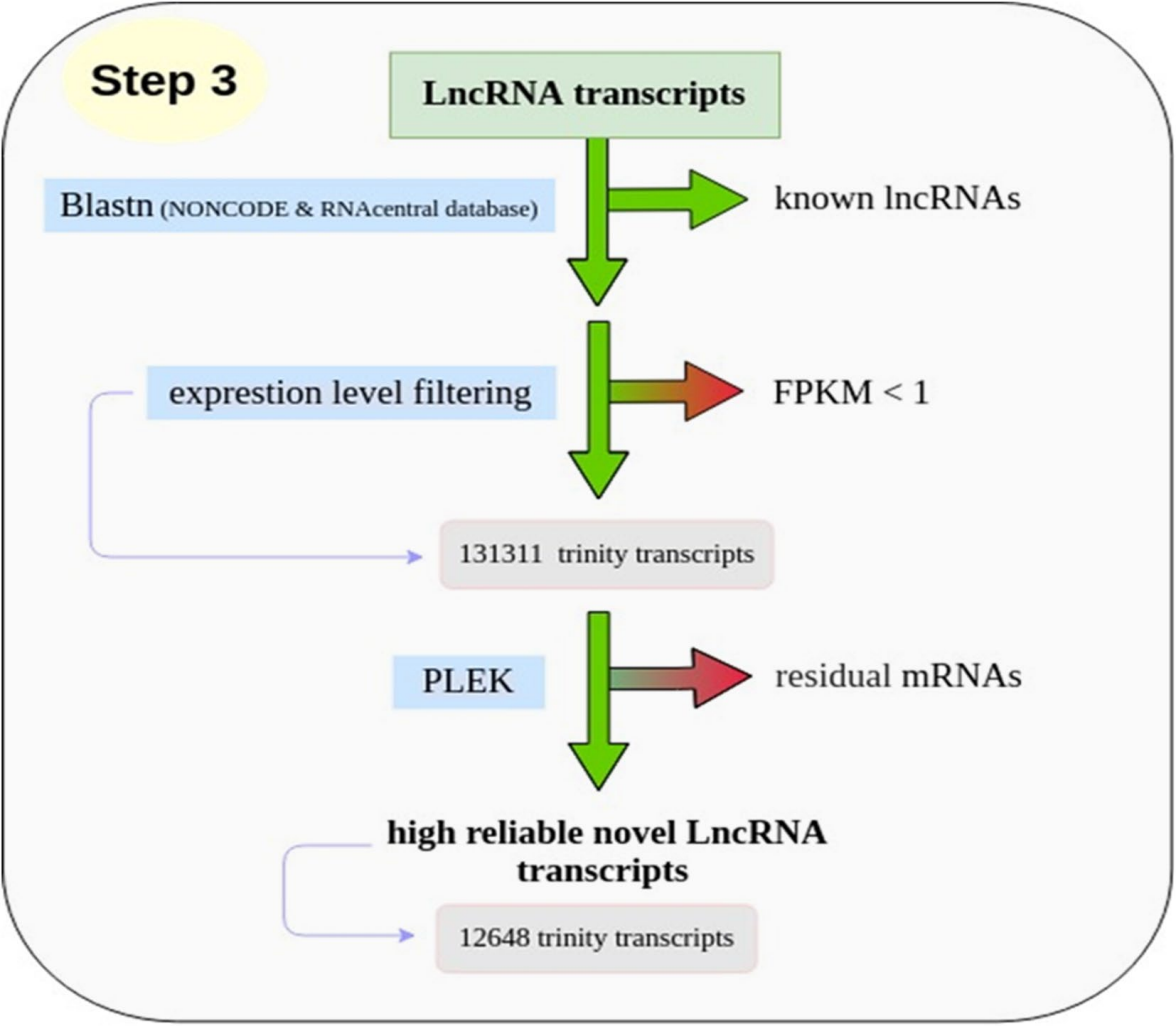
Overview of experimental and computational filtering pipeline (ECF). ECF pipeline is composed of computational and experimental steps: (1) Identification of ncRNAs, (2) Annotation and classification of ncRNAs, (3) Prediction of high reliable lncRNAs. Briefly, the cleaned reads were assembled using Trinity and then evaluated for protein coding portability with coding potential calculator 2 (CPC2). A series of protein annotations were performed using BLASTX and Pfam. The remaining ncRNA transcripts were filtered based on coding probability ≥ 0.4, transcript length ≤   300 nt and, open reading frames (ORFs) ≥ 300nt. INFERNAL and RNACentral were used to classify ncRNAs into various ncRNA families. The housekeeping ncRNAs also were removed. Transcripts that passed all criteria steps of ECF pipeline were classified as lncRNAs. In addition, RNACentral and NONCOD databases were used to predict the known lncRNAs. Finally, transcripts remaining after application of various filtering steps based on FPKM < 1 and PLEK, were known as novel set of high confidence transcripts.

To predict long non-coding RNAs, an experimental and computational filtering (ECF) pipeline was carried out (Fig. 1). The main steps of the ECF pipeline are similar to previously reported procedures^20,21^. LncRNA discovery approaches show similarities among different studies (Additional file 1). The procedure is as follows:

1. CPC2 software was used to score for coding potential^22^. Besides, this tool searches the sequences against the protein database and distinguishes protein-coding from non-coding RNAs. The CPC2 was tested on its web server because the web server usually performs better (Additional file 2). In the CPC2 tool, lncRNAs were shown as non-coding RNAs longer than 200 nt. Out of 952,725 transcripts, 47,982 were shown to be coding by CPC2, and they were removed.
2. The remaining 904,743 transcripts were then filtered due to coding potential. The CP threshold used for the scorpion dataset was 0.4. Scorpion transcripts with CP ≥ 0.4 were declared putatively coding and discarded, while those with CP score < 0.4 were retained as noncoding candidates.
3. To eliminate transcripts harboring any protein domains, we implemented various blast search methods; at first, remaining 901,937 transcripts were exposed to Swissprot database, NCBI non-redundant (Nr), and Pfam protein domain databases to find protein-coding transcripts. Blastx was used to search against non-redundant (Nr) and Swissprot databases with an *E*-value threshold of 10^−3^. Moreover, remaining scorpion (*A. crassicauda*) transcripts were searched for sequence similarity with Uniprot scorpion, tick, and spider protein sequences using blastx (*E*-value 1e−3). On the other hand, manually generated protein-toxin database, which includes all venom proteins and toxins sequence of venomous animal reviewed in Uniprot was used against remaining transcripts (*E*-value 1e−3). All 202,064 transcripts which returned at least one hit by one of used search procedures were discarded. So, 745,889 transcripts without coding potential was considered as ncRNA candidates for subsequent analyzes.
4. Three certain sequential stringent filters were performed to predict lncRNA candidates which included filtering due to transcript length, ORF size, and type of ncRNAs (e.g., housekeeping ncRNAs, microRNAs, etc.). For further details, transcript sequences shorter than 300 nucleotides were filtered out. Subsequently, for ORF determination, the remaining transcripts of this step, 387,637 ncRNA were loaded to the getorf website. Since known eukaryotic proteins have a length of more than 100 amino acids, this study similar to other studies, transcripts with an ORF of less than 300 nt have been classified as non-coding RNAs^23–25^. Ultimately, obtained transcripts were subjected to Rfam and RNACentral v14 databases.

**Figure 2:**
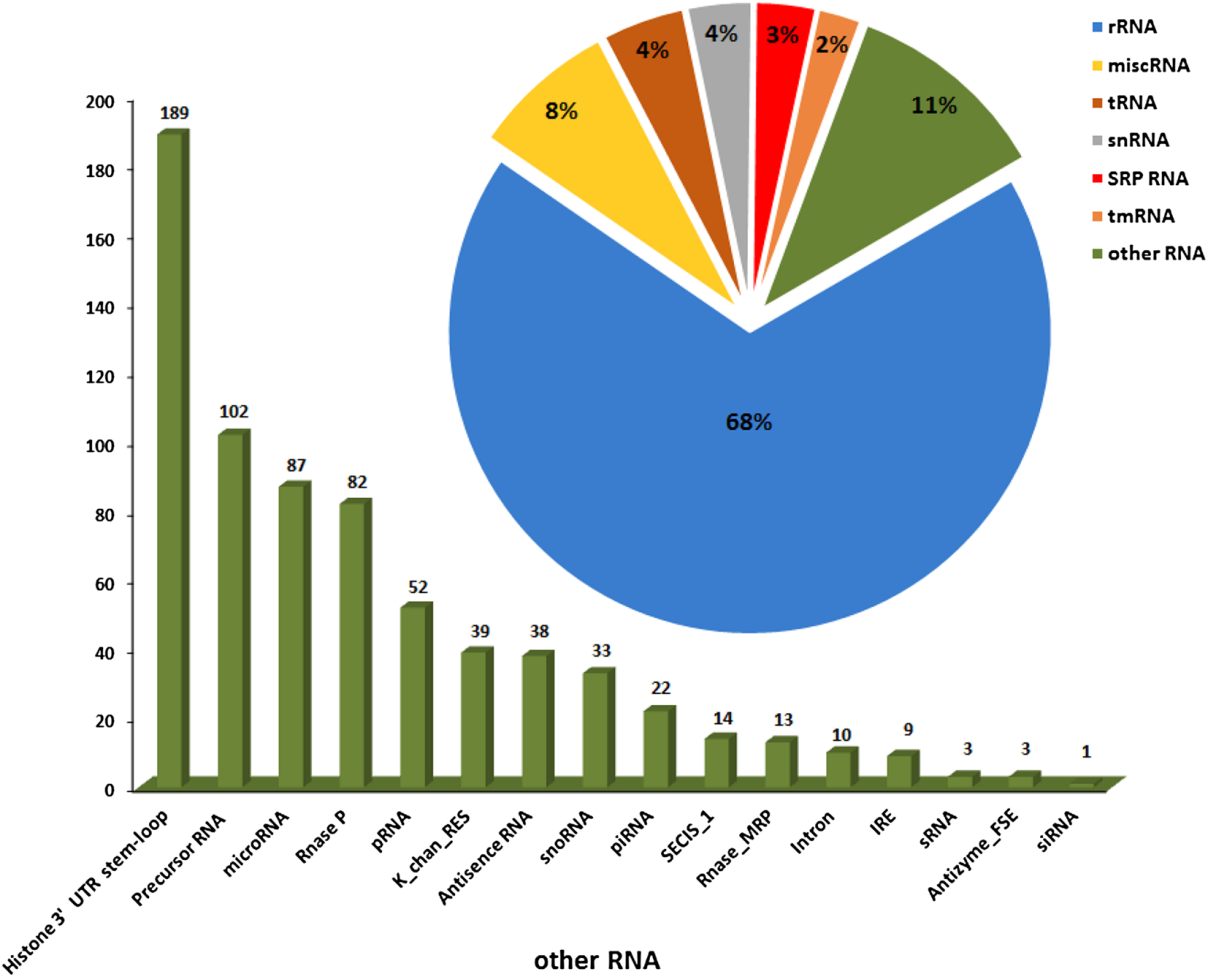
List of housekeeping and regulatory non-coding RNAs.

In more precisely, all obtained ncRNAs were classified into two categories: housekeeping and regulatory ncRNA transcript. The regulatory ncRNA also can be grouped as small non-coding RNAs and long non-coding RNA^26,27^. The list of housekeeping and regulatory ncRNAs obtained in this analysis is shown in Fig. 2. All discovered housekeeping ncRNAs and small ncRNAs from mentioned databases were removed from the dataset in this step. Therefore, a total of 367,332 transcripts were distinguished and introduced as the scorpion lncRNAs.

### Extract of known lncRNAs

To avoid loss of known lncRNAs, all identified scorpion lncRNAs were initially aligned with known lncRNA sequences of diverse species in RNACentral v14 and NONCODE v3.0 databases using blastn^28^. Any transcript with blast algorithm results in these databases with an *E*-value less than 0.00001 was considered known lncRNAs, and 368,991 retained lncRNAs were passed to the next filtering steps. The overlapping results indicated that despite the majority of predicted scorpion lncRNAs sequences (12,642 out of 13,401 transcripts) have no identifiable orthologs even in closely related species, 759 (5.7%) of these lncRNAs have homologs in other species. This result has been observed in other species^29^. The matching RNACentral v14 IDs and NONCODE IDs for each distinguished scorpion lncRNAs are listed in Additional file 3 and Additional file 4, respectively.

### LncRNA expression in scorpion venom gland

We calculated the expression values of lncRNAs in the scorpion venom glands using RSEM software. As shown by previous studies, lncRNAs are typically lower in expression level than protein-coding genes^30,31^; however, to exclude any transcriptional noise, lncRNAs with FPKM of less than 1 were dropped out. Using these steps, 131,311 putative scorpion-specific lncRNAs were used for further analysis.

### Evaluation of ECF pipeline predictive reliability

Finally, an efficient alignment-free computational tool named PLEK with default pre-built models was employed to increase the reliability of lncRNAs prediction, and only transcripts which were labeled as noncoding in output were remained with high confidence to be novel scorpion-specific lncRNAs. We ultimately got a set of 12,642 novel lncRNA transcripts corresponding to 11,039 genes. Current annotation listed 759 lncRNA transcripts; hence, the total number of lncRNAs in *A. crassicauda* was 13,401 transcripts. This Targeted Locus Study (TLS) project was deposited at DDBJ/EMBL/GenBank under the accession KEPY00000000, associated with the BioProject PRJNA687110 and biosample SAMN17133090. The version described in this paper is the first version, KEPY01000000.

### Performance of computational approaches on scorpion datasets

This study aims to introduce the best tool to predict ncRNAs and mRNAs. Due to the insufficient amount of experimentally validated ncRNAs in arachnida, there is no specific computational program to stimulate ncRNAs in these species. To find the best software, four computational programs, PLEK, CNIT, CPC, and Annocript, were implemented using total assembled scorpion transcripts, and their results were compared.

CPC2 is a fast predictor of coding potential which uses a support vector machine due to ORF length, Fickett score, ORF integrity, and isoelectric point to differentiate coding and noncoding RNAs^32^. Using CPC2 web server for ncRNA prediction, we select the fruit fly as the appropriate species model. Using 952,725 de novo assembled transcripts, 904,743 ncRNAs and a set of 47,982 protein-coding transcripts were obtained.

PLEK uses a computational pipeline due to SVM algorithm and an improved k-mer scheme to distinguish ncRNAs from mRNAs^17^. It employed the model trained on the human database to predict the sequences of invertebrates. In this work, 911,471 and 40,503 transcripts were identified to be noncoding and mRNA in PLEK algorithms, respectively.

Annocript, a pipeline for annotating de novo assembled transcriptome, is established to combine the annotation of protein-coding transcripts with predicting putative lncRNAs. Although it has a model for all organisms in Uniprot, which can be customized by users, all organisms were definitely chosen. This program executes following analysis: Blastx against TrEMBL/UniRef and Swissprot, RPSBLAST against CDD profiles, BLASTN against Rfam and rRNAs, dna2pep and Portrait software to extract many features and classified the transcripts as lncRNA due to transcript length (> 200 nt), ORF (< 300 nt), non-coding potential score (≥ 0.95) and transcripts with no match in public databases^33^. We predicted a set of 122,421 mRNA and 5955 known lncRNAs using the Annocript platform.

CNIT (Coding-Non-Coding Identifying Tool) software is a powerful tool to effectively distinguish between protein-coding and non-coding sequences by profiling adjoining nucleotide triplets ANT due to sequence intrinsic composition. CNIT has models for animals and plants. Among all assembled transcripts, 904,112 transcripts were classified as non-coding RNAs, while 48,613 transcripts were protein-coding. The overall performance of ECF pipeline and four ncRNA prediction tools are displayed in Table 1.

**Table 1:** Performance of lncRNA identification tools and ECF pipeline on whole *Androctonus crassicauda* dataset.

Programs	Protein coding		Non coding		Known lncRNA		Novel lncRNA	
	Isoform	Gene	Isoform	Gene	Isoform	Gene	Isoform	Gene
PLEK	40,503	25,814	911,471	570,142	–	–	–	–
CPC2	47,982	17,141	904,743	579,021	–	–	–	–
Annocript	122,421	53,227	15,467	10,560	5955	5161	–	–
CNIT	48,613	20,058	904,112	575,634	–	–	–	–
ECF pipeline	202,064	92,031	13,399	11,724	759	687	12,642	11,039

Due to Table 1, Annocript, despite having a long wait presented the best result than CPC, CNIT, and PLEK. During comparing results, we realized that CPC2 and CNIT software had almost the same results and enabled to predict lncRNAs the same as the PLEK, while ECF pipeline and Annocript display reasonable results with lncRNA prediction. The initial comparison (Table 1) shows that the highest protein values were identified by the ECF pipeline, followed by Annocript, while the lowest predicted protein values were obtained by PLEK software, compared to noncoding results. Annocript exhibited much higher known lncRNAs prediction.

**Figure 3:**
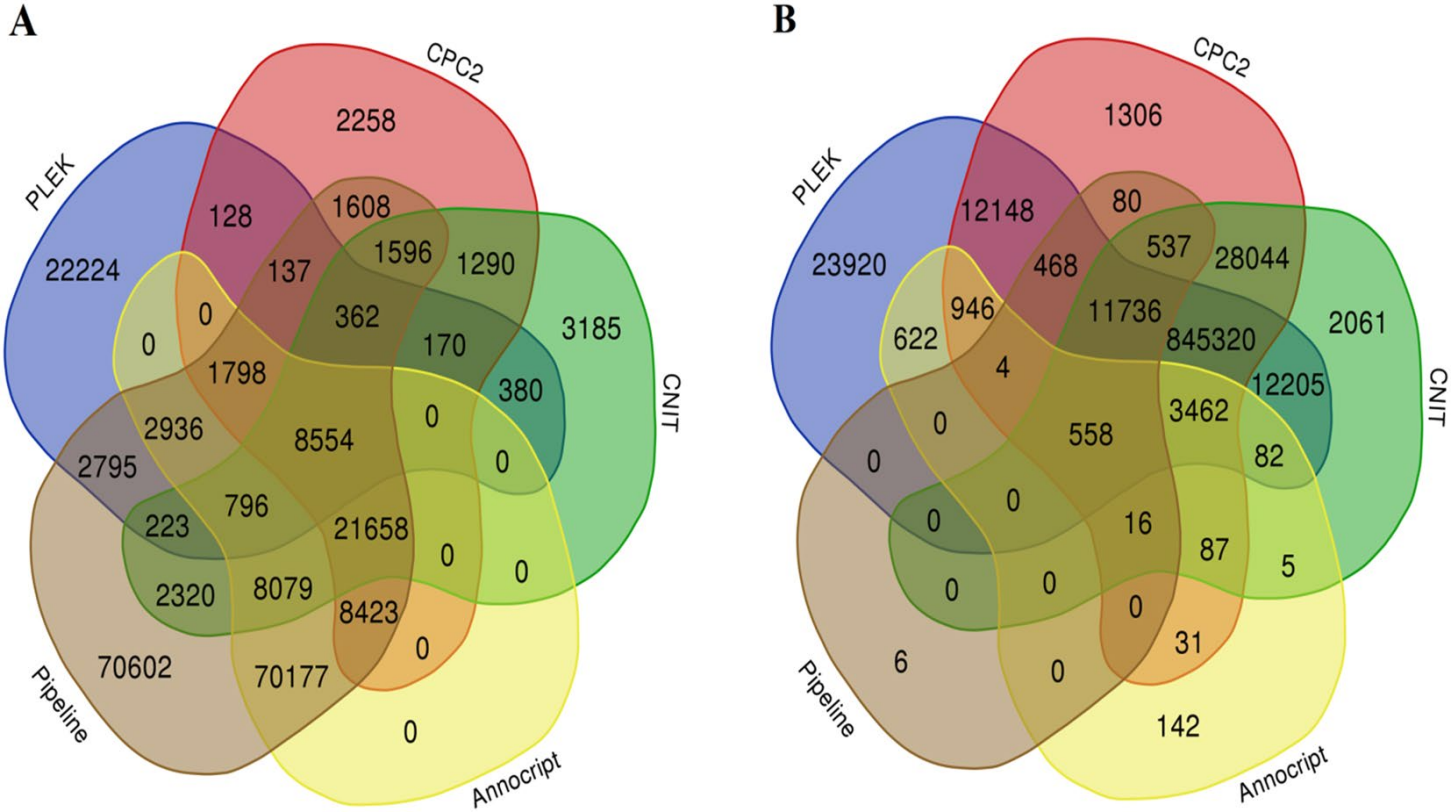
Performances of PLEK, CPC, CNIT and Annocript on scorpion dataset. (**A**) The fraction of scorpion dataset classified as protein-coding transcripts. (**B**) The fraction of scorpion dataset classified as non-coding transcripts.

Furthermore, Venn diagrams (http://bioinformatics.psb.ugent.be/webtools/Venn) were utilized to plot the performance of lncRNA prediction tools visually (Fig. 3). Venn diagram exhibit that not only all the mRNAs predicted with Annocript overlap with other approaches, almost 98% of its predicted lncRNAs also overlap with others (Fig. 3). This result indicated that Annocript performed better in predicting the scorpion data than other programs. Besides, due to Fig. 3, it seems that there is a significant coding or non-coding PLEK misclassified transcripts. Although there is a high overlap between the ECF pipeline and other tools, the highest unique mRNA and the lowest unique lncRNA have appeared in the ECF pipeline. This observation suggests that nevertheless ECF approach performed very strict to identify novel lncRNAs; it is able to detect more proteins-coding sequences, it means ECF pipeline presents an outstanding performance on the scorpion data set which offers a great application prospect to the analysis of arthropods transcripts.

**Figure 4:**
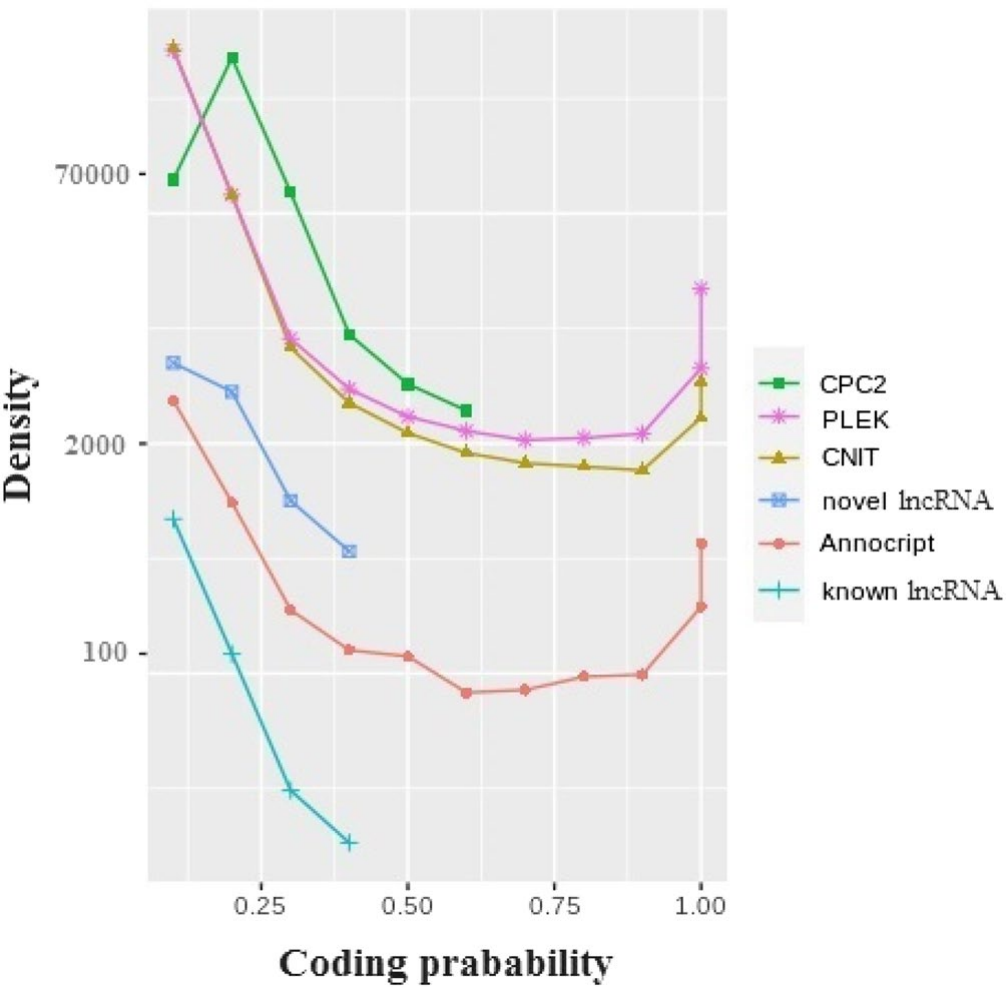
Coding probability distribution of predicted ncRNAs using CPC2, PLEK, CNIT, Annocript and, ECF pipeline.

Comparison of predicted coding probability, it can be a good assessment of lncRNA prediction tool performance. This comparison indicates that novel lncRNAs predicted using the ECF pipeline have a lower coding probability threshold, even slightly lower than predicted known lncRNAs. In contrast, PLEK and CNIT, followed by Annocript (Fig. 4), exhibited the highest CP score. Annocript was executed with default parameters; thus, the 0.95 coding probability thresholds were used as cut-off which can be changed by user.

### Evaluation of the sensitivity and the specificity

**Figure 5:**
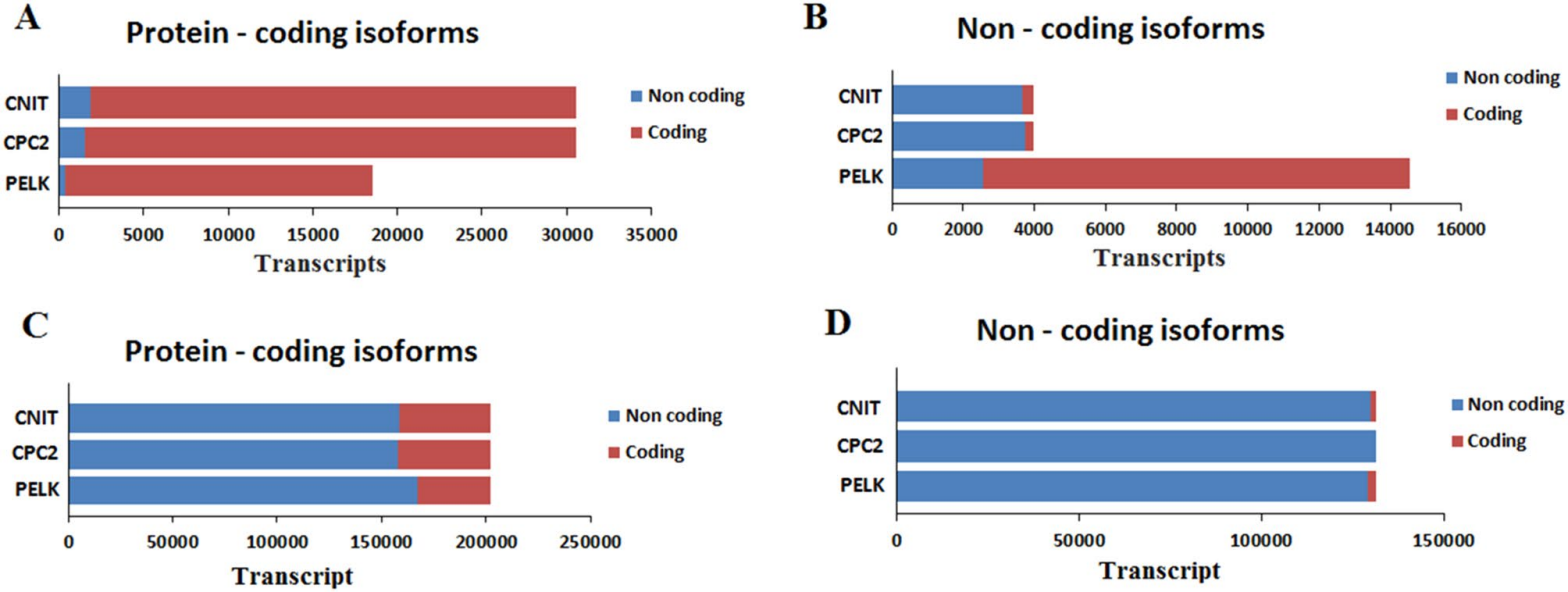
Results of computational approaches on fruit fly and scorpion datasets. (**A**,**B**) Classification of protein-coding and non-coding transcripts of fruit fly using CNIT, CPC2 and, PLEK tools. (**C**,**D**) Classification of protein-coding and non-coding transcripts of scorpion using CNIT, CPC2 and PLEK software programs.

To evaluate the sensitivity and specificity of the ECF pipeline and four popular lncRNA prediction tools on the scorpion dataset, as a model of arachnida species, once again, PLEK, CNIT, CPC2, and ECF pipeline were done by utilizing the 131,311 lncRNAs and 202,064 mRNAs scorpion dataset of this study. Furthermore, we compared ECF pipeline's performance with that of PLEK, CNIT, and CPC using a test dataset, which includes 3976 lncRNAs and 30,588 mRNAs of the fruit fly. Detailed information of datasets was summarized in the methods section. Figure 5 showed that using lncRNAs prediction tools in species without closely related organisms to build ncRNA/mRNA distinguishing model increases the false positive rate compared to database derived dataset. As a comparison, at least 6.19, 8.07 and 9.45% of fruit fly non-coding dataset were misclassified as coding by CPC, CNIT and PLEK respectively (Fig. 5B), while the scorpion dataset were used, 1.03, 0 and 15.91% of non-coding transcripts were misclassified as coding by CNIT, CPC, and PLEK, respectively (Fig. 5D).

For data collected from the database, CPC and CNIT showed good performance as compared with PLEK (Table 2). In contrast, predicted results for the scorpion dataset are not satisfactory and almost similar compared to each other (Table 3). From Tables 2 and 3, we can find that the ECF pipeline achieved a balanced overall result with high accuracy. In detail, CPC2 achieved a satisfactory result (sensitivity: 0.94, specificity: 0.95, accuracy: 0.95) on the fruit fly dataset (Table 2), which was higher than that of CNIT (sensitivity: 0.92, specificity: 0.94, accuracy: 0.94) and PLEK (sensitivity: 0.87, specificity: 0.60, accuracy: 0.63). CPC2 indicated high positive and negative predictive values 0.71 and 0.99, respectively, on fruit fly dataset. Nonetheless, PLEK had relatively better NPV 0.98 but poor PPV 0.18. ECF pipeline achieved the highest accuracy of 0.99, specificity 1, sensitively 0.91, PPV 1, and NPV 0.99 on the fruit fly dataset. Moreover, the ECF pipeline correctly predicts 92.38% (3673/3976) lncRNAs and 100% (30,588/30,588) mRNAs for fruit fly testing dataset. While, CPC2, PLEK and CNIT were applied on the scorpion dataset, the accuracy values are 0.53, 0.49, and 0.52, respectively (Table 3).

**Table 2:** Performance of lncRNA identification tools and ECF pipeline on *Drosophila melanogaster* dataset.

Programs	Sensitivity	Specificity	Accuracy	PPV	NPV
PLEK	0.87	0.60	0.63	0.18	0.98
CPC2	0.94	0.95	0.95	0.71	0.99
CNIT	0.92	0.94	0.94	0.66	0.99
ECF pipeline	0.91	1	0.99	1	0.99

**Table 3:** Performance of lncRNA identification tools and ECF pipeline on selected *Androctonus crassicauda* dataset.

Programs	Sensitivity	Specificity	Accuracy	PPV	NPV
PLEK	0.98	0.17	0.49	0.44	0.94
CPC2	1	0.22	0.53	0.45	1
CNIT	0.99	0.22	0.52	0.45	0.97
ECF pipeline	1	1	1	1	1

Sensitivity, specificity and accuracy were calculated using the formulae mentioned in methods and listed in this table.

**Figure 6:**
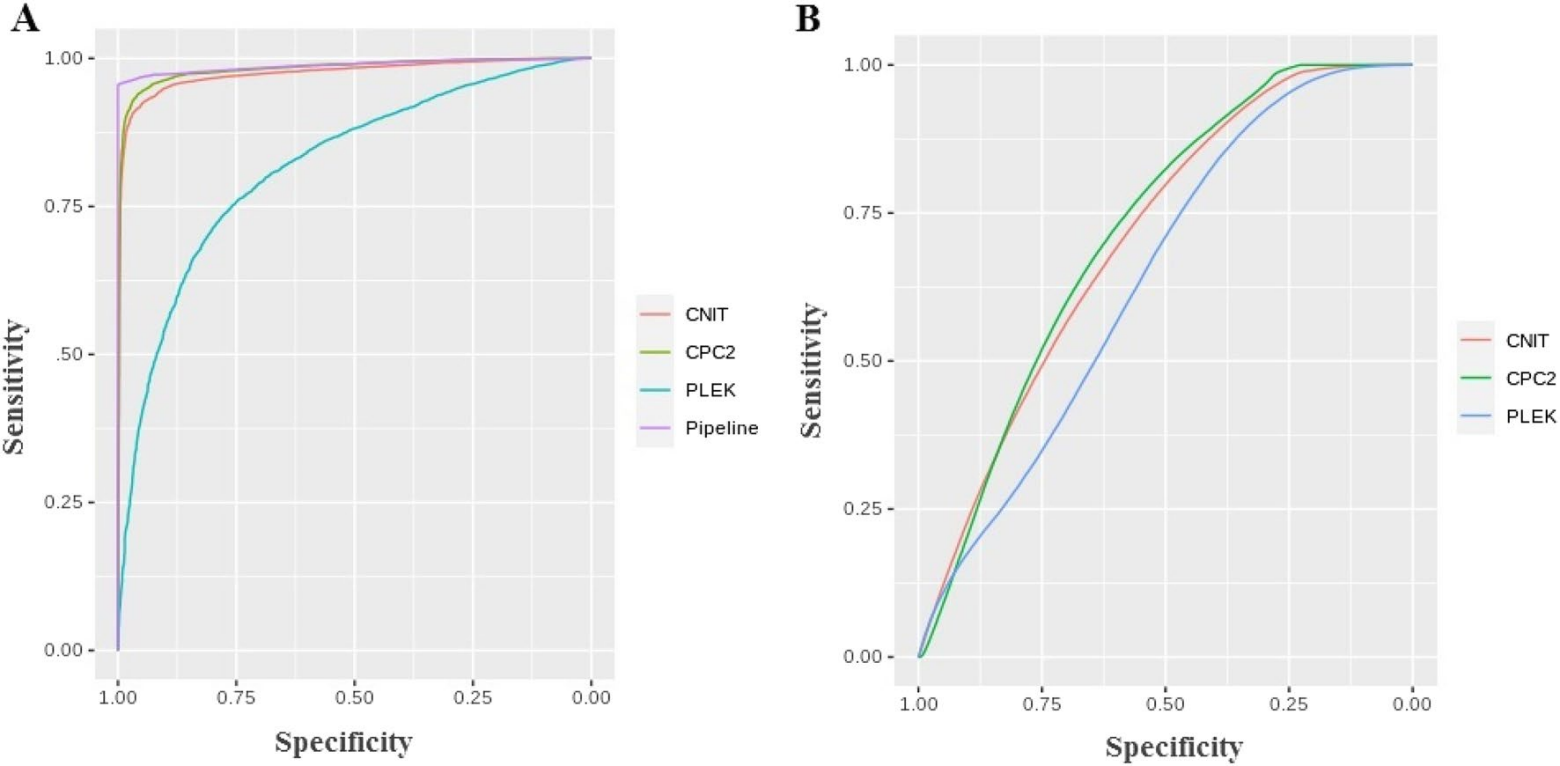
Sensitivity and specificity comparison of ECF pipeline with software programs (CNIT, CPC2 and PLEK) on: (**A**) *Drosophila melanogaster* datasets (**B**) *Androctonus crassicauda* RNA-seq datasets. The Area Under the Curve (AUC) measures the performance of an algorithm under different thresholds.

Finally, we generate a ROC curve to visualize the classification performances of four approaches further (Fig. 6). From Fig. 6A, we note that the ECF pipeline, CPC2, and CNIT struck a good balance between sensitivity and specificity as well as obtaining a reasonable result. Nonetheless, the PLEK tool presented a sharp decline in specificity. An area under the receiver operating characteristic (AUC) curve visualized in Fig. 6 indicates better insight into the performance of approaches to separate two classes. From the fruit fly and scorpion datasets, a higher AUC was observed for ECF pipeline and CPC2 respectively.

### Characterization analyses of lncRNA

**Figure 7:**
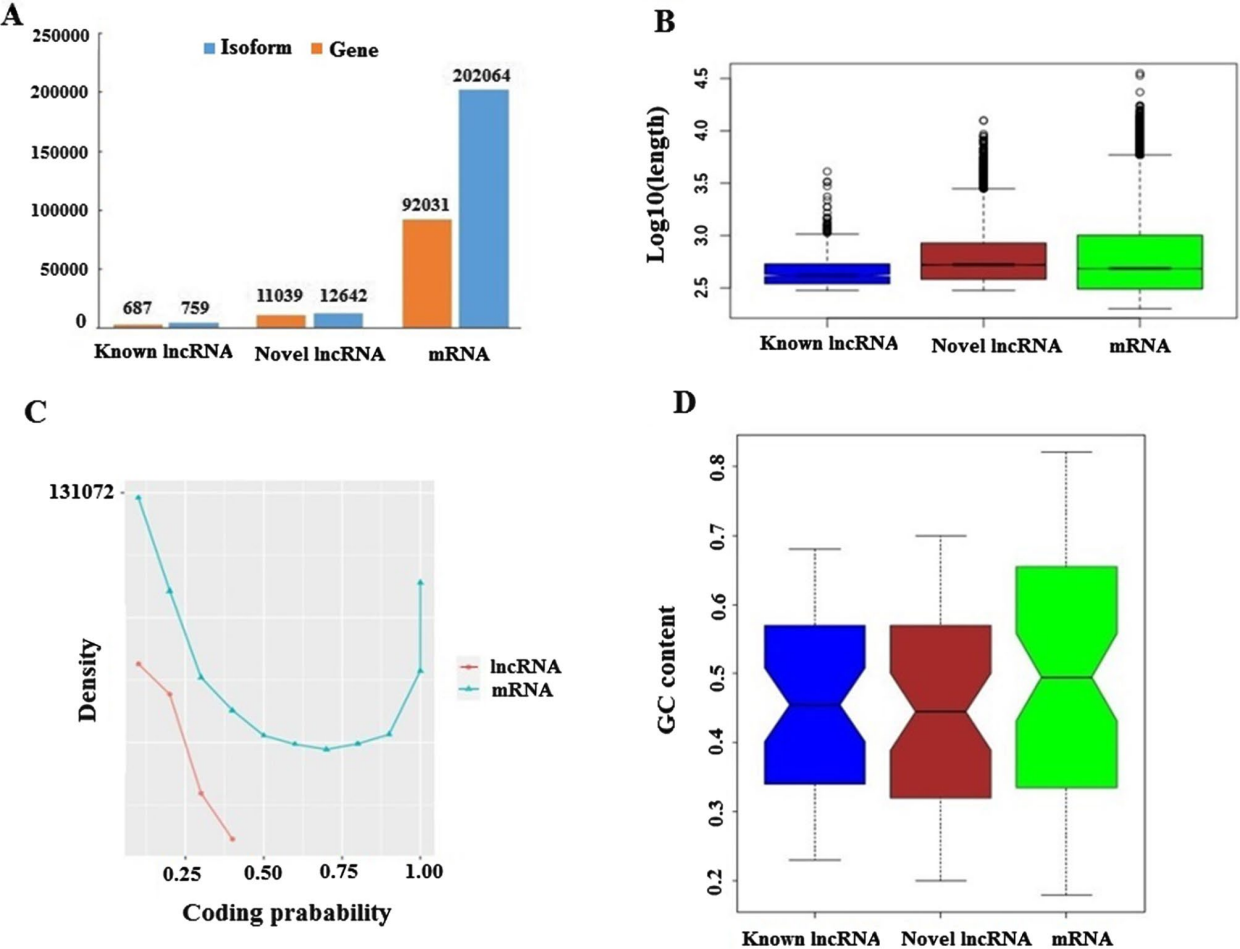
Characterization of *A. crassicauda* lncRNA. (**A**) Number of isoforms per gene (**B**) Length distribution of known lncRNA, novel lncRNA and, mRNA transcripts (**C**) Coding probability of known lncRNAs, novel lncRNAs and, mRNAs (**D**) GC content of known lncRNA, novel lncRNA and, mRNA transcripts.

To analyze whether the main characteristics of *A*. *crassicauda* lncRNAs typical exhibit features observed in previous studies^12,27,34^, the features of predicted lncRNAs transcripts were compared to protein-coding transcripts, isoform per gene, coding probability, GC content and sequence length (Fig. 7). We realized that almost all known and novel lncRNAs had an average of 1.1 isoforms per gene, while protein coding genes having more than 2 isoforms per gene (Fig. 7A). Similar to previous reports, lncRNA transcripts harbored a lower isoforms than protein-coding gene^21,34^.

In agreement with the main characteristics described in the studies done in other species^21,34–37^, our data suggested that lncRNA transcripts were on average shorter than protein-coding RNAs (Fig. 7B). Novel and known lncRNAs had a mean length of 762.2 bp and 504.15 bp respectively, while the average length of protein-coding transcripts was 871.9 bp.

Early studies have strongly emphasized the inability of lncRNA to code the proteins^38^, so we evaluated the coding probability of our identified lncRNAs and compared them with protein coding transcripts. We found that, our predicted lncRNA transcripts exhibited lower coding probability than that of protein-coding transcripts (Fig. 7C). Moreover, analysis of the novel lncRNAs indicated a low GC content (42.6%), similar to what was observed in known lncRNAs (43.4%), which is significantly lower than protein coding sequence (50.8%) (Fig. 7D).

## Discussion

By the special role of lncRNAs in regulating gene expression, controlling various biological processes, and cellular functions^3,5,7^, their identification which leads to the discovery of many sophisticate mechanisms of gene regulation has become important in different species. Development of high-throughput sequencing cooperated with bioinformatics tools, has aid lncRNAs uncover in many insect species^4,12,13^.

Various lncRNA prediction pipelines were described in detail in non-model animals; while the predictions of lncRNAs of the vast majority of arthropods remain elusive^20,21^. To date, many studies were done on scorpion transcriptomes^9,14,39^; however, none of them identified the scorpion-specific lncRNA. The present work provided the analysis on scorpion venom gland lncRNAs which have not been studies to date. This study used high-throughput sequencing technology combined with bioinformatics for detection of lncRNA transcripts in scorpion venom gland. In addition to high accuracy lncRNA prediction pipeline, we provided most comprehensive dataset of scorpion lncRNAs, which is consist of multiple information of scorpion lncRNAs, like expression profile, coding probability distribution, features of predicted lncRNAs, annotation, etc.

Currently, identifying lncRNAs from mRNAs in arthropod, especially in species without a reference genome faces various challenges. To overcome this problem, we tried two methods of predicting lncRNAs. (1) We tested various developed machine learning-based tools to detect the scorpion lncRNAs. (2) We designed a filtering pipeline to identify novel and known lncRNAs.

Nowadays, various machine learning-based approaches were developed to facilitate and speed up the lncRNA prediction^15–17,33^.

Following trained PLEK, CNIT, Annocript, and CPC2 approaches in scorpion data set, we evaluated the performance of approaches. Due to insufficient scorpion lncRNAs, the predicted scorpion data set along with fruit fly lncRNA/mRNA data set were used to evaluate the sensitivity and specificity of approaches.

In this work have shown that Annocript be a powerful platform for the identification of scorpion lncRNA transcripts in high-throughput sequencing data. In Annocript, users can easily customize lncRNAs features to enhance the sensitivity and specificity of lncRNAs prediction models on different species. Although the performance of other examined tools in the scorpion-specific data set was not satisfactory, the predictive performance of CPC2 was higher over other approaches. Due to the results of previous experiments^1,40^ and this study obtained results, we conclude that computational lncRNA prediction tools are not the specific tool to predict lncRNAs in species without reference genomes or with insufficient annotated protein-coding sequences. Part of the reasons for these outcomes are the lack of conservation among lncRNA primary sequences, insufficient lncRNA information of many species, and relatively low association of computational analysis tools to diverse databases dedicated to lncRNA research^1,41–43^. Moreover, most machine learning-based tools for lncRNAs prediction often utilize only animal training data sets and cannot be user-adjustable for different species^43,44^.

More detection of invertebrate lncRNAs through targeted experiments, it eventually enhances the predictive performance of lncRNAs tools. Therefore, judging the performance of software based only on a few data collected from databases is not justifiable because working with large-scale data, especially for species without reference genomes or closely related organisms to build models to distinguish their ncRNA and mRNA transcripts, changes all equations^1^. It is now highly recommended to use step-by-step filtering pipeline instead of lncRNA computational prediction programs to identify the lncRNAs of these organisms.

Nevertheless, to reduce the false-positive rate by improving the specificity and sensitivity, we performed a ECF pipeline to identify novel and known lncRNAs that simultaneously uses two lncRNA prediction tools to calculate the coding probability of protein (CPC2) and assess whether the predicted transcripts are indeed likely to be non-coding RNAs (PLEK). The obtained results indicated that the ECF pipeline is suited for de novo assembled data sets from scorpion species. Thus, this ECF pipeline helps distinguish protein-coding and non-coding transcripts from RNA sequencing data of many arthropod species without reference genomes. Identifying novel lncRNAs greatly increases the knowledge of arthropod ncRNAs.

Aside from identifying the scorpion lncRNAs, ECF pipeline will be useful to characterize lncRNAs from deep sequencing data. As shown in previous reports, this type of studies revealed fundamental features of lncRNAs in vertebrate and invertebrate animals, including their low GC content as well as coding probability, shorter length sequence, and less number of isoform per gene^21,36,45–47^. Numerous studies have reported that lncRNAs play a wide range of structural and regulatory roles in key biological processes. Accumulating evidence suggests that some aspects of lncRNA function depend on the structural properties of RNA molecules; hence it is important to indicate the sequence properties of lncRNAs. The unveiling of distinctive features of lncRNA not only serves to distinguish lncRNAs from other RNAs in non-model species but may also help to improve predictions of their functional mechanisms in the future. Interestingly, Previous published studies have suggested that the short length, lower GC content, lower average level of expression, and lower cellular level of lncRNAs compared with the protein-coding RNAs, could potentially mean that lncRNAs sequences are less stable than protein-coding mRNAs, which this, in turn, may explain some aspects of lncRNA function, such as their ability to fold into different structures and to conduct molecular interactions with other cellular factors^47–50^.

Similar to previous studies, analysis of lncRNA characteristics in *A. crassicauda* uncovered that lncRNAs shared strikingly similar features with other species. The trend of lower protein-coding potential, lower GC content, shorter transcript length, and less number of isoform per gene in lncRNAs sequence over protein-coding transcripts are outstanding features of *A. crassicauda* lncRNAs transcripts that mean these sequences contain lower stably base-paired structures and therefore, it is more possible to interact with other cellular factors^21,37,45–47^.

## Materials and methods

### RNA extraction and de novo transcriptome assembly

The *A. crassicauda* specimens were collected from Baghmalek, Khozestan providence southwest of Iran. All captured scorpions were taxonomically identified according to Koch^51^, quickly milked and maintained in a plastic box with water and crickets ad libitum for 3 days. subsequently, scorpions venom gland were powdered with a porcelain mortar and pestle under liquid nitrogen and total RNA extraction was performed using RNeasy Animal Mini Kit (Qiagen, Valencia, CA, USA) according to the manufacturer’s instructions. Finally, all samples were sequenced with 150 bp paired-end reads at Macrogen Co (Macrogen, Seoul*,* South Korea) using Illumina HiSeq 2000 sequencing platform (Illumina, San Diego, CA, USA). The raw sequences and clean data were subjected to FastQC for quality assessment of sequences (Supplementary figures F1–F6).

After filtering, cleaning and trimming of the raw reads generated from Illumina sequencing platform, clean reads were de novo assembled into contigs using Trinity software (v. 2.0.3)^18^ with optimized parameters.

### Pipeline for identification of lncRNAs

Step-by-step experimental and computational filtering (ECF) pipeline was used to minimize the false positives rate of lncRNAs prediction. Also additional annotation programs including CPC2 (coding potential calculator software based on alignment-based algorithms, version 2.0) and PLEK (predictor of long non-coding RNAs and messenger RNAs based on an improved k-mer scheme, version 1.2) were employed. Both of these techniques make it possible to identify more accurate lncRNAs (Fig. 1).

### Computational identification of protein-noncoding transcripts using CPC2

Initially, all assembled transcripts were subjected to CPC2 to evaluate their coding potential^32^. Then in order to distinguish ncRNAs from protein-coding transcripts, we focused on transcripts labeled as “noncoding” in the output and filtered out any transcripts that had higher coding probability. As suggested in recent studies, the optimum cut-off for protein coding probability (CP) varies depending on the species^22^ and setting a high coding probability threshold, leads to increase the misclassified transcripts as non-coding or coding^52^. Therefore, in species with no specified coding threshold such as scorpion, it is best to use studies of closely related species.

In this regard, to make error probability as small as possible, coding probability threshold of ECF pipeline was set at ≥ 0.4, based on the specified CP threshold of fruit fly^22^. ECF pipeline basically filters out any transcript with high coding potential, which estimated with CPC2. The transcripts scored with a probability less than 0.4 were considered noncoding-RNA candidates.

### Annotation of all non-redundant transcripts

For annotation of assembled transcripts, the remaining ncRNA candidates were submitted to blastx search with an *E* value threshold le−3 against the following databases: Swissprot (A manually annotated and reviewed protein sequence database); Nr (NCBI non-redundant protein sequences); UniProtKB/TrEMBL and, Pfam (Protein family). In purpose of discarding any known protein domain, we employed species-specific annotation. In summary, scorpion-specific annotation consists of three steps: (1) Downloading the specific sequences of scorpion, tick, spider (https://www.uniprot.org/) and, all manually reviewed venom proteins and toxins from the venomous animals (https://www.uniprot.org/program/Toxins) in fasta format. (2) Building customized databases with local sequences by means of the makeblastdb. (3) Annotation using blastx. Thereafter, all positively annotated transcripts were discarded from lncRNA candidates.

### Filtering and classification of putative ncRNAs transcripts

To extract reliable putative ncRNAs, we set the minimum assembled transcript length to longer than 300 bp and those that were ≤ 300 bp in length were removed. After that, the remaining transcripts were subjected to getorf website (http://www.bioinformatics.nl/cgi-bin/emboss/getorf) to find longest ORFs, and those transcripts with ORF longest than ≥ 300 nt were also discarded.

The remaining transcripts were then subjected to a Rfam database to exclude any housekeeping and small RNAs, such as tRNAs, rRNAs, snRNAs, snoRNAs, micro-RNA, piRNA, siRNA and, other RNAs (*E*-value < 0.001) using BLASTN. To ensure that housekeeping and small RNAs were removed from the putative ncRNA dataset, we performed blastn against RNACentral db (http://rnacentral.org) to find and discard housekeeping RNA residuals. The remaining transcripts were considered as large non-coding RNAs.

### Novel lncRNA prediction

Known lncRNA sequences including all validated lncRNAs were downloaded from two lncRNA databases: (1) NONCODE database (http://www.noncode.org/). (2) RNACentral release 14 (http://rnacentral.org). Overlap of scorpion lncRNAs with these lncRNA database sources was determined using blastn with a cut-off *E*-value of 10^**–**3^. Then, known lncRNAs were extracted from whole lncRNAs list and the analysis was continued with the novel lncRNAs.

### Transcript expression

Gene expression levels in terms of FPKM were quantified using RSEM software^53^. Extremely low gene expression is generally considered to be transcriptional noise^54^. To enhance the reliability of our prediction, we set an FPKM (fragments per kilobase of transcript per million fragments assembled) value of 1 as the lower bound in subsequent analyses and any input transcripts with FPKM value greater than 1 were included in high reliable lncRNA list.

### Prediction of high reliable novel lncRNA

To detect high reliable novel lncRNAs, the remaining transcripts were subjected to PLEK tool^17^. PLEK is a developed computational software to distinguish lncRNAs from mRNAs in RNA-seq transcriptomes of species lacking reference genomes (https://sourceforge.net/projects/plek/files/).

### Computational identification of lncRNA in scorpion datasets

In addition to using ECF pipeline for predicting scorpion lncRNAs, we tested various lncRNA prediction tools on whole assembled scorpion dataset which include, CPC2, PLEK, CNIT (http://cnit.noncode.org/CNIT/) and Annocript (https://github.com/frankMusacchia/Annocript) with the default parameters. Venn diagram (http://bioinformatics.psb.ugent.be/webtools/Venn/) was used to visualize the resulting data. Coding potential assessment was performed for all approaches and the scatter curve was drawn to compare the coding probability of identified ncRNAs, known lncRNAs and novel lncRNAs.

### Data collection and description

We finally compared ECF pipeline with lncRNA prediction tools using data sets of scorpion and fruit fly. Considering lack of lncRNA genomic coordinates for scorpion, the approaches were trained and tested on *drosophila melanogaster* dataset retrieved from NONCODE and Ensamble databases, which contains 3976 lncRNAs and 30,588 mRNA sequences. In addition, scorpion lncRNAs and mRNA transcripts that were predicted in this study were used to assess the programs performance on scorpion dataset, which contains 131,311 lncRNAs and 202,064 mRNAs.

### Comparative analysis

Sensitivity, specificity, accuracy (ACC), positive predictive value (PPV) and, negative predictive value (NPV) metrics were used to assess classification performance of the computational programs on d*rosophila melanogaster* and *Androctonus crassicauda* datasets. In order to intuitively measure the performance, the ROK curves were employed.

$$Accuracy=\frac{TP+TN}{TP+FP+FN+TN}$$

$$Specificity=\frac{TN}{FP+TN}$$

$$Sensitivity=\frac{TP}{TP+FN}$$

$$PPV=\frac{TP}{TP+FP}$$

$$NPV=\frac{TN}{TN+FN}$$

TP means true positive, FN refer to false negative, FP is false positive, and TN represents true negative.

### Characterization analyses of lncRNA

Characterization of coding and long non-coding RNAs was determined using number of isoform per gene, protein coding probability, length, and, GC content assays. Meanwhile, GC content of each sequence was evaluated using EMBOSS geecee program. Ultimately, Sequence length, GC content and CP distribution of lncRNA and mRNA were plotted.

### Ethical statement

The manuscript and data were not previously or simultaneously submitted elsewhere. All experiments in this paper were carried out under the standard procedures of scientific ethics, including the care of experimental animals. All authors have read the manuscript and agree to its publication in Journal of Scientific Report and agree that it has followed the rules of ethics presented in the guidelines for journal publication.

## Supplementary Information

Supplementary Information 1.

Supplementary Information 2.

Supplementary Information 3.

Supplementary Information 4.

Supplementary Information 5.
