# Supplementary material for: Systematic and computational identification of Androctonus crassicauda long non-coding RNAs
Source: Sci Rep. 2021 Feb 25;11:4720. doi: 10.1038/s41598-021-83815-8 (PMC7907363; doi:10.1038/s41598-021-83815-8)
Supplement: Supplementary file 1 — Supplementary Information 1. [file 41598_2021_83815_MOESM1_ESM.docx]

**Additional file 1**

**Systematic and computational identification of *Androctonus Crassicauda* long non-coding RNAs**

**Salabi Fatemeh^1^, Jafari Hedieh^1^, Navidpour Shahrokh^2^, Sadr Ayeh Sadat^3^**

1-Razi Vaccine and Serum Research Institute, Agricultural Research, Education and Extension Organization (AREEO), Ahvaz, Iran. 2. Razi Vaccine and Serum Research Institute, Agricultural Research, Education and Extension Organization (AREEO), Karaj, Iran. 3. Aquaculture Research Center-South of Iran, Iranian Fisheries Science Research Institute, Agricultural Research, Education and Extension Organization (AREEO), Ahvaz, Iran.

***Corresponding author:** Fatemeh Salabi, Department of Venomous Animals and Anti-venom Production, Razi Vaccine and Serum Research Institute, Agricultural Research, Education and Extension Organization (AREEO), Ahvaz, Iran. Telephone and Fax Numbers: 0098-613332504

Email: f.salabi@rvsri.ac.ir

**Name: Fatemeh Salabi**

Address: Department of Venomous Animals and Anti-venom Production, Razi Vaccine and Serum Research Institute, Agricultural Research, Education and Extension Organization (AREEO), Ahvaz, Iran. Telephone and Fax Numbers: 0098-613332504

Email: f.salabi@rvsri.ac.ir

URL: Razi Vaccine and Serum Research Institute, Agricultural Research, Education and Extension Organization (AREEO), Ahvaz, Iran.

**Name: Hedieh Jafari**

Address: Department of Venomous Animals and Anti-venom Production, Razi Vaccine and Serum Research Institute, Agricultural Research, Education and Extension Organization (AREEO), Ahvaz, Iran. Telephone and Fax Numbers: 0098-613332504

Email: hedieh_jafari@yahoo.com

URL: Razi Vaccine and Serum Research Institute, Agricultural Research, Education and Extension Organization (AREEO), Ahvaz, Iran.

**Name: Shahrokh Navidpour**

Address: Department of Venomous Animals and Anti-venom Production, Razi Vaccine and Serum Research Institute, Agricultural Research, Education and Extension Organization (AREEO), Karaj, Iran. Telephone and Fax Numbers: 0098-613332504

Email ID: navid1038@hotmail.com

**Name: Ayeh Sadat Sadr**

Address: Aquaculture Research Center-South of Iran, Iranian Fisheries Science Research Institute, Agricultural Research Education and Extension Organization (AREEO), Ahvaz, Iran.

Email: ayehsadr@gmail.com

**Additional file 1.** Comparison of ECF pipeline with three studies that have predicted lncRNAs using RNA-seq.

| Reference | Organism | Cell/tissue | RNA-seq library type ^a^ | Assembly | Size selection ^b^ | Coding potential | Annotation | ncRNAs classification | Genome reference |
| --- | --- | --- | --- | --- | --- | --- | --- | --- | --- |
| ECF pipeline | Scorpion | Venom gland | rRNAd | de novo assembly (Trinity) | 300 nt | CPC2<0.4, ORF<100 aa, BLASTX, BLASTP & PLEK | Swissprot, NCBI Nr, UniProtKB/TrEMBL, Pfam, SignalP, scorpion specific annotation, Rfam, NONCODE & RNAcentral | **√** | **×** |
| Azlan , et al., 2019 | mosquito | Cell line | Total RNA | de novo assembly (Stringtie) | 200 nt | ORF<0 ^c^, CPAT<0.3& BLASTX | Gffcompare & Swissprot | **×** | **√** |
| Huang, et al., 2017 | anthozoan | Colonies | pA^+^ | de novo assembly (Trinity) | 200 nt | BLASTX, ORF<75 aa, & PLEK | NCBI Nr, Pfam, SignalP & Rfam | **√** | **×** |
| Wang, et al., 2018 | diamondback moth | Eggs, larvae, adults | Total RNA | Cufflinks | 200 nt | CPC<0, CNCI<0, Cpat<0.39, BLASTX & ORF<100 aa |  | **×** | **√** |

**a** Removal of rRNAs from a sample is commonly achieved either by selecting only RNA molecules that contain a poly-A tail (pA+), or by specifically depleting rRNA (rRNAd).

**b** Size selection of assembled transcript.

**c** Those having ORF were discarded.
