# Supplementary material for: Systematic and computational identification of Androctonus crassicauda long non-coding RNAs
Source: Sci Rep. 2021 Feb 25;11:4720. doi: 10.1038/s41598-021-83815-8 (PMC7907363; doi:10.1038/s41598-021-83815-8)

**Additional file 2**

**Systematic and computational identification of *Androctonus Crassicauda* long non-coding RNAs**

**Salabi Fatemeh^1^, Jafari Hedieh^1^, Navidpour Shahrokh^2^, Sadr Ayeh Sadat^3^**

1-Razi Vaccine and Serum Research Institute, Agricultural Research, Education and Extension Organization (AREEO), Ahvaz, Iran. 2. Razi Vaccine and Serum Research Institute, Agricultural Research, Education and Extension Organization (AREEO), Karaj, Iran. 3. Aquaculture Research Center-South of Iran, Iranian Fisheries Science Research Institute, Agricultural Research, Education and Extension Organization (AREEO), Ahvaz, Iran.

***Corresponding author:** Fatemeh Salabi, Department of Venomous Animals and Anti-venom Production, Razi Vaccine and Serum Research Institute, Agricultural Research, Education and Extension Organization (AREEO), Ahvaz, Iran. Telephone and Fax Numbers: 0098-613332504

Email: f.salabi@rvsri.ac.ir

**Name: Fatemeh Salabi**

Address: Department of Venomous Animals and Anti-venom Production, Razi Vaccine and Serum Research Institute, Agricultural Research, Education and Extension Organization (AREEO), Ahvaz, Iran. Telephone and Fax Numbers: 0098-613332504

Email: f.salabi@rvsri.ac.ir

URL: Razi Vaccine and Serum Research Institute, Agricultural Research, Education and Extension Organization (AREEO), Ahvaz, Iran.

**Name: Hedieh Jafari**

Address: Department of Venomous Animals and Anti-venom Production, Razi Vaccine and Serum Research Institute, Agricultural Research, Education and Extension Organization (AREEO), Ahvaz, Iran. Telephone and Fax Numbers: 0098-613332504

Email: hedieh_jafari@yahoo.com

URL: Razi Vaccine and Serum Research Institute, Agricultural Research, Education and Extension Organization (AREEO), Ahvaz, Iran.

**Name: Shahrokh Navidpour**

Address: Department of Venomous Animals and Anti-venom Production, Razi Vaccine and Serum Research Institute, Agricultural Research, Education and Extension Organization (AREEO), Karaj, Iran. Telephone and Fax Numbers: 0098-613332504

Email ID: navid1038@hotmail.com

**Name: Ayeh Sadat Sadr**

Address: Aquaculture Research Center-South of Iran, Iranian Fisheries Science Research Institute, Agricultural Research Education and Extension Organization (AREEO), Ahvaz, Iran.

Email: ayehsadr@gmail.com

**Additional file 2.** Comparative performance of CPC2 on its stand-alone version and web server using scorpion datasets. (A) The performance of CPC on scorpion protein-coding dataset. (B) The performance of CPC on scorpion non-coding dataset.


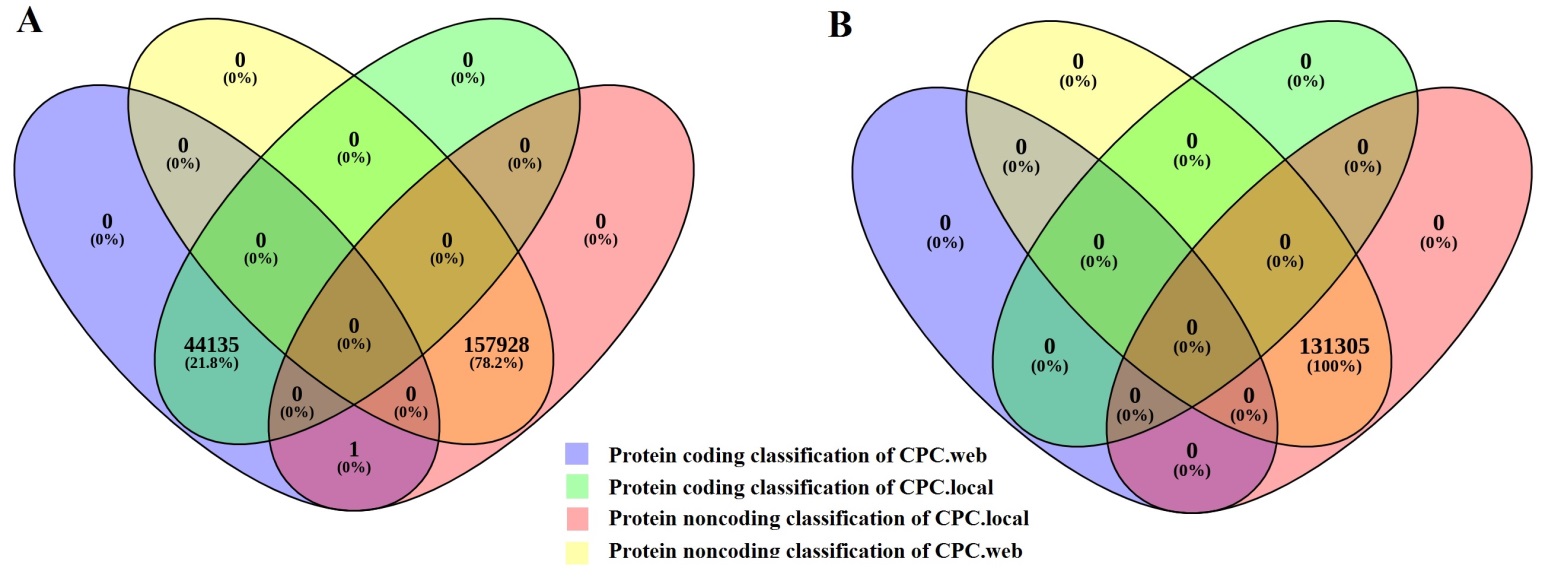

Supplement: Supplementary file 2 — Supplementary Information 2. [file 41598_2021_83815_MOESM2_ESM.docx]
