# Supplementary material for: Systematic and computational identification of Androctonus crassicauda long non-coding RNAs
Source: Sci Rep. 2021 Feb 25;11:4720. doi: 10.1038/s41598-021-83815-8 (PMC7907363; doi:10.1038/s41598-021-83815-8)
Supplement: Supplementary file 3 — Supplementary Information 3. [file 41598_2021_83815_MOESM3_ESM.docx]

**Additional file 3**

**Systematic and computational identification of *Androctonus Crassicauda* long non-coding RNAs**

**Salabi Fatemeh^1^, Jafari Hedieh^1^, Navidpour Shahrokh^2^, Sadr Ayeh Sadat^3^**

1-Razi Vaccine and Serum Research Institute, Agricultural Research, Education and Extension Organization (AREEO), Ahvaz, Iran. 2. Razi Vaccine and Serum Research Institute, Agricultural Research, Education and Extension Organization (AREEO), Karaj, Iran. 3. Aquaculture Research Center-South of Iran, Iranian Fisheries Science Research Institute, Agricultural Research, Education and Extension Organization (AREEO), Ahvaz, Iran.

***Corresponding author:** Fatemeh Salabi, Department of Venomous Animals and Anti-venom Production, Razi Vaccine and Serum Research Institute, Agricultural Research, Education and Extension Organization (AREEO), Ahvaz, Iran. Telephone and Fax Numbers: 0098-613332504

Email: f.salabi@rvsri.ac.ir

**Name: Fatemeh Salabi**

Address: Department of Venomous Animals and Anti-venom Production, Razi Vaccine and Serum Research Institute, Agricultural Research, Education and Extension Organization (AREEO), Ahvaz, Iran. Telephone and Fax Numbers: 0098-613332504

Email: f.salabi@rvsri.ac.ir

URL: Razi Vaccine and Serum Research Institute, Agricultural Research, Education and Extension Organization (AREEO), Ahvaz, Iran.

**Name: Hedieh Jafari**

Address: Department of Venomous Animals and Anti-venom Production, Razi Vaccine and Serum Research Institute, Agricultural Research, Education and Extension Organization (AREEO), Ahvaz, Iran. Telephone and Fax Numbers: 0098-613332504

Email: hedieh_jafari@yahoo.com

URL: Razi Vaccine and Serum Research Institute, Agricultural Research, Education and Extension Organization (AREEO), Ahvaz, Iran.

**Name: Shahrokh Navidpour**

Address: Department of Venomous Animals and Anti-venom Production, Razi Vaccine and Serum Research Institute, Agricultural Research, Education and Extension Organization (AREEO), Karaj, Iran. Telephone and Fax Numbers: 0098-613332504

Email ID: navid1038@hotmail.com

**Name: Ayeh Sadat Sadr**

Address: Aquaculture Research Center-South of Iran, Iranian Fisheries Science Research Institute, Agricultural Research Education and Extension Organization (AREEO), Ahvaz, Iran.

Email: [ayehsadr@gmail.com](mailto:ayehsadr@gmail.com)

**Additional file 3. RNACentral v14 IDs for each distinguished scorpion lncRNAs have homologs in other species.**

| URS0000203D46 |
| --- |
| URS000031C6A7 |
| URS000038D830 |
| URS000048A512 |
| URS00004B8B40 |
| URS00004CB04A |
| URS000054AABB |
| URS000057896B |
| URS0000759B65 |
| URS0000759E9B |
| URS000075A44C |
| URS000075A6BC |
| URS000075A72B |
| URS000075AA7D |
| URS000075AB50 |
| URS000075AE75 |
| URS000075B2E7 |
| URS000075B437 |
| URS000075B993 |
| URS000075C817 |
| URS000075C907 |
| URS000075CEFB |
| URS000075CF3E |
| URS000075D1FD |
| URS000075D2DA |
| URS000075D386 |
| URS000075DCEF |
| URS000075E18C |
| URS000075E9F2 |
| URS000075EC38 |
| URS000075F8E5 |
| URS0000760EB7 |
| URS0000761B00 |
| URS0000763E50 |
| URS00007663CB |
| URS0000766FE5 |
| URS000076714D |
| URS000076B5DA |
| URS000076BCFE |
| URS00007708DC |
| URS0000771425 |
| URS0000772030 |
| URS0000774888 |
| URS00007758E7 |
| URS000077594C |
| URS0000777E66 |
| URS00007788BD |
| URS000077BD77 |
| URS000077F72E |
| URS000077FE55 |
| URS0000780A46 |
| URS0000781890 |
| URS0000782079 |
| URS0000782FCD |
| URS0000783E1F |
| URS000078482C |
| URS00007853BA |
| URS00007859AD |
| URS0000785FC6 |
| URS0000786921 |
| URS0000786E69 |
| URS000078762C |
| URS0000787933 |
| URS0000787E94 |
| URS0000787EB0 |
| URS0000788020 |
| URS0000788A84 |
| URS0000788D95 |
| URS0000788EA3 |
| URS0000788EBB |
| URS00007892AF |
