# Supplementary material for: Systematic and computational identification of Androctonus crassicauda long non-coding RNAs
Source: Sci Rep. 2021 Feb 25;11:4720. doi: 10.1038/s41598-021-83815-8 (PMC7907363; doi:10.1038/s41598-021-83815-8)

**Supplementary Figures**

**Systematic and computational identification of *Androctonus Crassicauda* long non-coding RNAs**

**Salabi Fatemeh^1^, Jafari Hedieh^1^, Navidpour Shahrokh^2^, Sadr Ayeh Sadat^3^**

1-Razi Vaccine and Serum Research Institute, Agricultural Research, Education and Extension Organization (AREEO), Ahvaz, Iran. 2. Razi Vaccine and Serum Research Institute, Agricultural Research, Education and Extension Organization (AREEO), Karaj, Iran. 3. Aquaculture Research Center-South of Iran, Iranian Fisheries Science Research Institute, Agricultural Research, Education and Extension Organization (AREEO), Ahvaz, Iran.

***Corresponding author:** Fatemeh Salabi, Department of Venomous Animals and Anti-venom Production, Razi Vaccine and Serum Research Institute, Agricultural Research, Education and Extension Organization (AREEO), Ahvaz, Iran. Telephone and Fax Numbers: 0098-613332504

Email: f.salabi@rvsri.ac.ir

**Name: Fatemeh Salabi**

Address: Department of Venomous Animals and Anti-venom Production, Razi Vaccine and Serum Research Institute, Agricultural Research, Education and Extension Organization (AREEO), Ahvaz, Iran. Telephone and Fax Numbers: 0098-613332504

Email: f.salabi@rvsri.ac.ir

URL: Razi Vaccine and Serum Research Institute, Agricultural Research, Education and Extension Organization (AREEO), Ahvaz, Iran.

**Name: Hedieh Jafari**

Address: Department of Venomous Animals and Anti-venom Production, Razi Vaccine and Serum Research Institute, Agricultural Research, Education and Extension Organization (AREEO), Ahvaz, Iran. Telephone and Fax Numbers: 0098-613332504

Email: hedieh_jafari@yahoo.com

URL: Razi Vaccine and Serum Research Institute, Agricultural Research, Education and Extension Organization (AREEO), Ahvaz, Iran.

**Name: Shahrokh Navidpour**

Address: Department of Venomous Animals and Anti-venom Production, Razi Vaccine and Serum Research Institute, Agricultural Research, Education and Extension Organization (AREEO), Karaj, Iran. Telephone and Fax Numbers: 0098-613332504

Email ID: navid1038@hotmail.com

**Name: Ayeh Sadat Sadr**

Address: Aquaculture Research Center-South of Iran, Iranian Fisheries Science Research Institute, Agricultural Research Education and Extension Organization (AREEO), Ahvaz, Iran.

Email: ayehsadr@gmail.com

**Figure F1. Sequencing quality report of RNA-seq of *Androctonus Crassicauda* datasets by Fastqc.** A) Sequence length distribution of raw reads from mature male *A. Crassicauda*. B) Per base quality of raw reads from mature male *A. Crassicauda*. C) Per sequence quality of raw reads from mature male *A. Crassicauda*. D) Per base n content of raw reads from mature male *A. Crassicauda*.


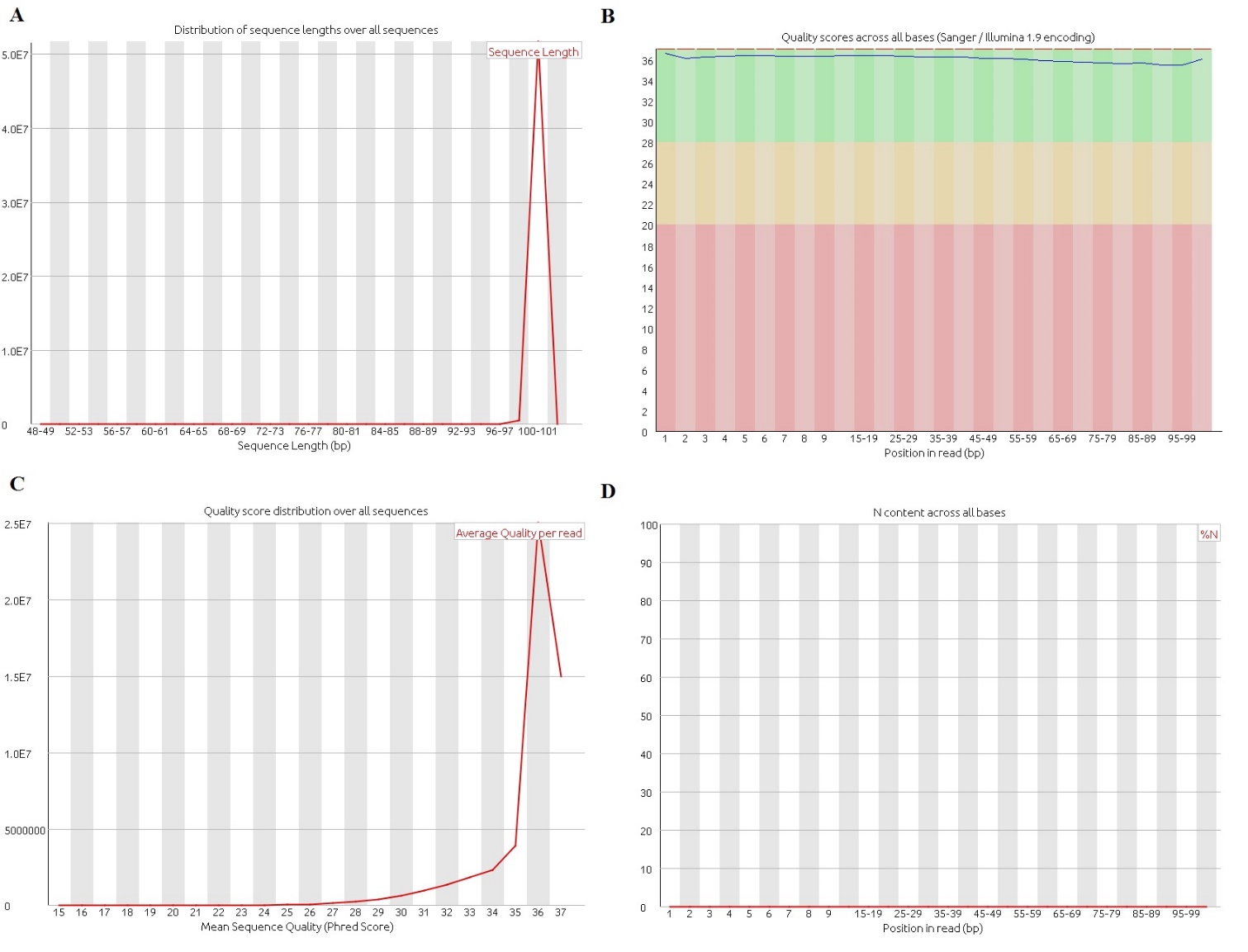


**Figure F2. Sequencing quality report of RNA-seq of *Androctonus Crassicauda* datasets by Fastqc.** A) Sequence length distribution of raw reads from immature male *A. Crassicauda.* B) Per base quality of raw reads from immature male *A. Crassicauda*. C) Per sequence quality of raw reads from immature male *A. Crassicauda*. D) Per base n content of raw reads from immature male *A. Crassicauda*.


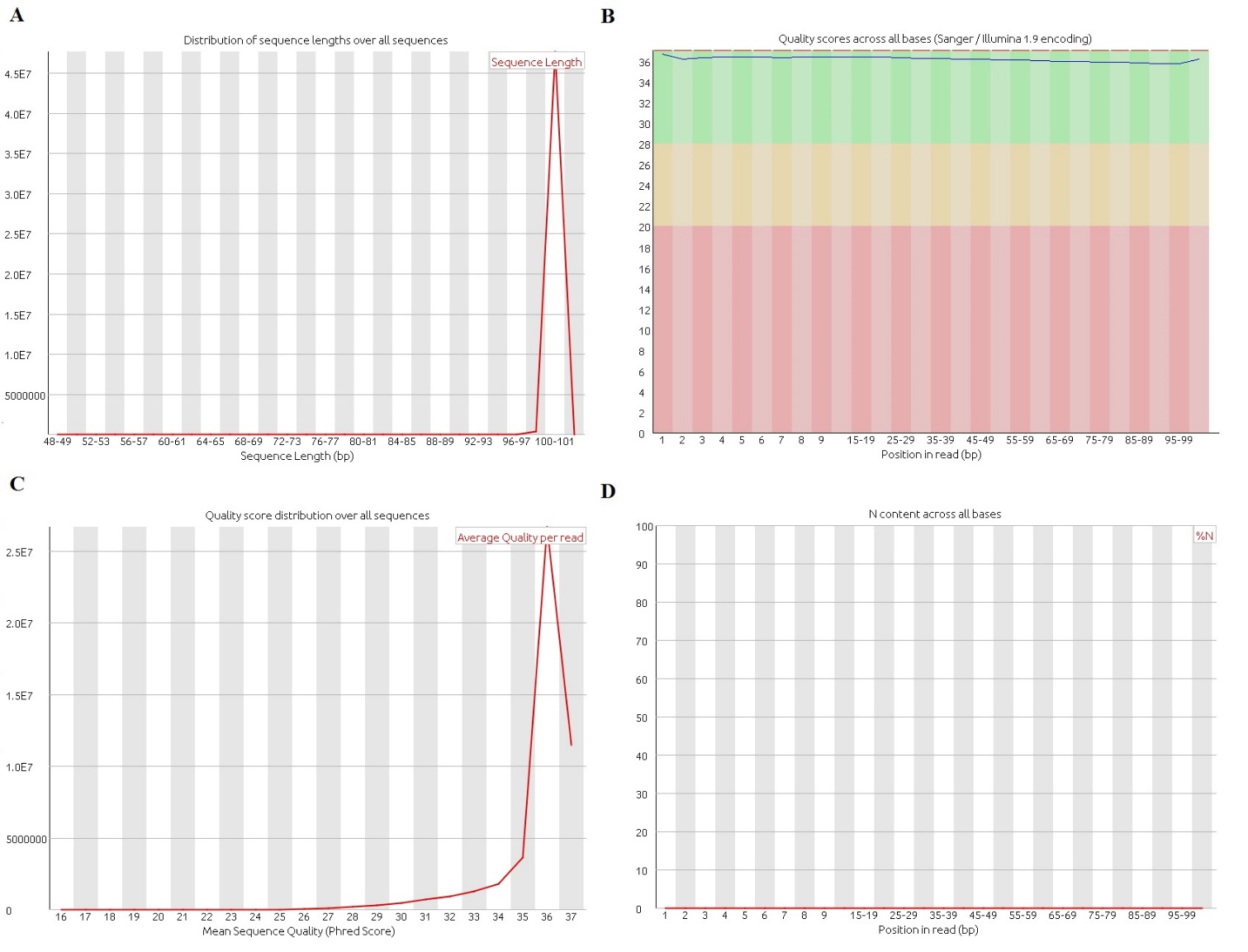


**Figure F3. Sequencing quality report of RNA-seq of *Androctonus Crassicauda* datasets using Fastqc.** A) Sequence length distribution of raw reads from immature male *A. Crassicauda.* B) Per base quality of raw reads from immature male *A. Crassicauda*. C) Per sequence quality of raw reads from immature male *A. Crassicauda*. D) Per base n content of raw reads from immature male *A. Crassicauda*.


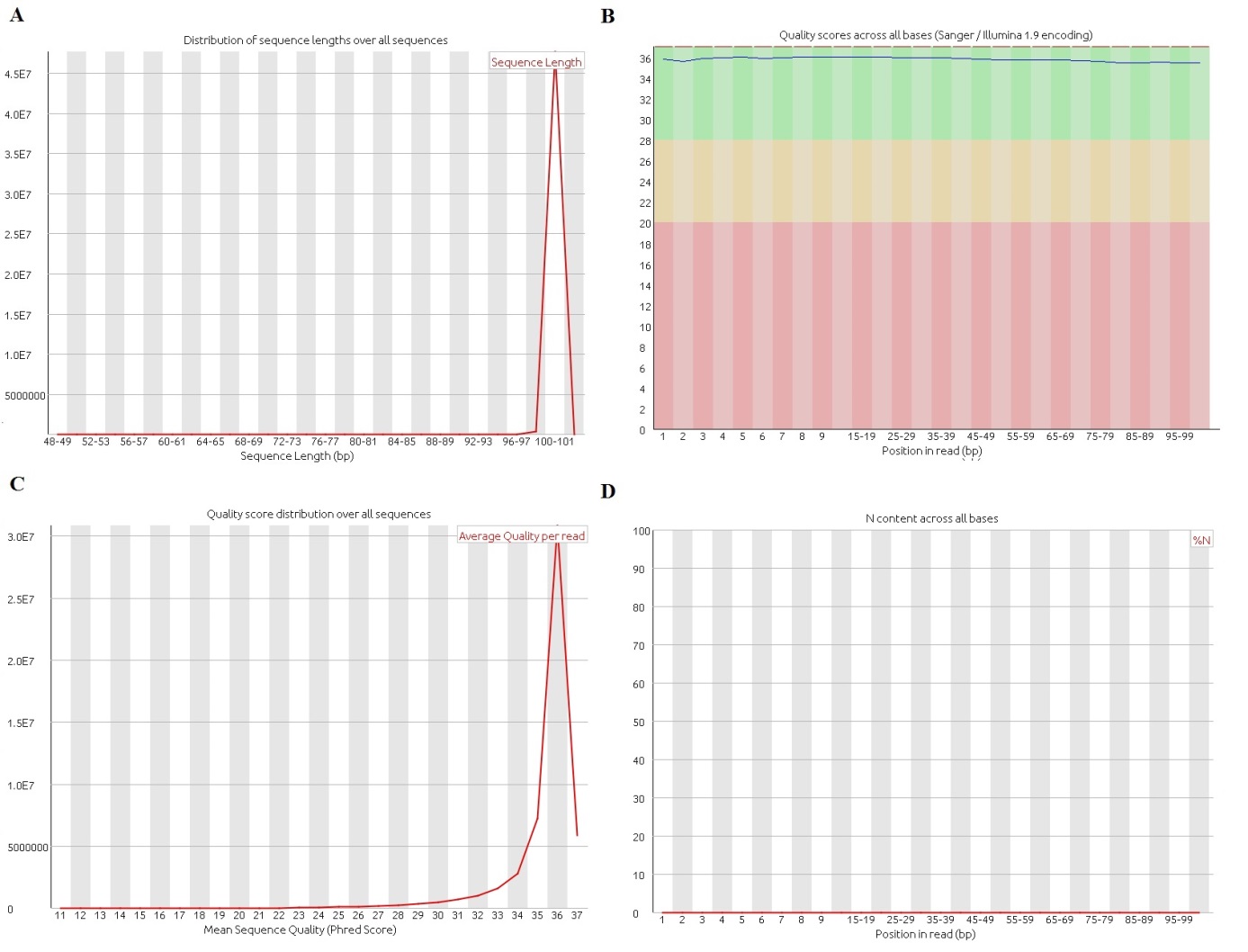


**Figure F4. Sequencing quality report of RNA-seq of *Androctonus Crassicauda* datasets by Fastqc.** A) Sequence length distribution of raw reads from mature female *A. Crassicauda*. B) Per base quality of raw reads from mature female *A. Crassicauda*. C) Per sequence quality of raw reads from mature female *A. Crassicauda*. D) Per base n content of raw reads from mature female *A. Crassicauda*.


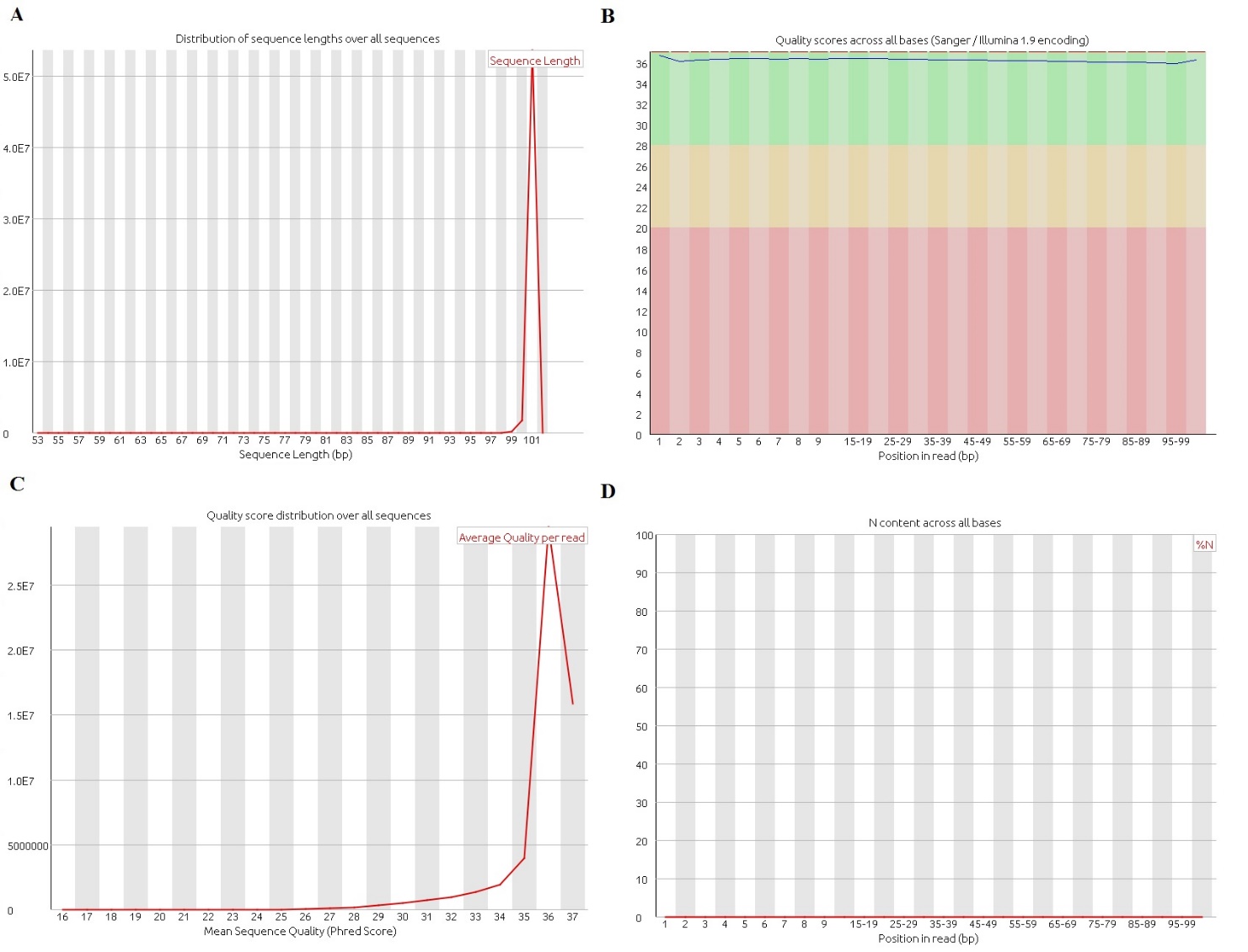


**Figure F5. Sequencing quality report of RNA-seq of *Androctonus Crassicauda* datasets by Fastqc.** A) Sequence length distribution of raw reads from immature female *A. Crassicauda*. B) Per base quality of raw reads from immature female *A. Crassicauda*. C) Per sequence quality of raw reads from immature female *A. Crassicauda*. D) Per base n content of raw reads from immature female *A. Crassicauda*.


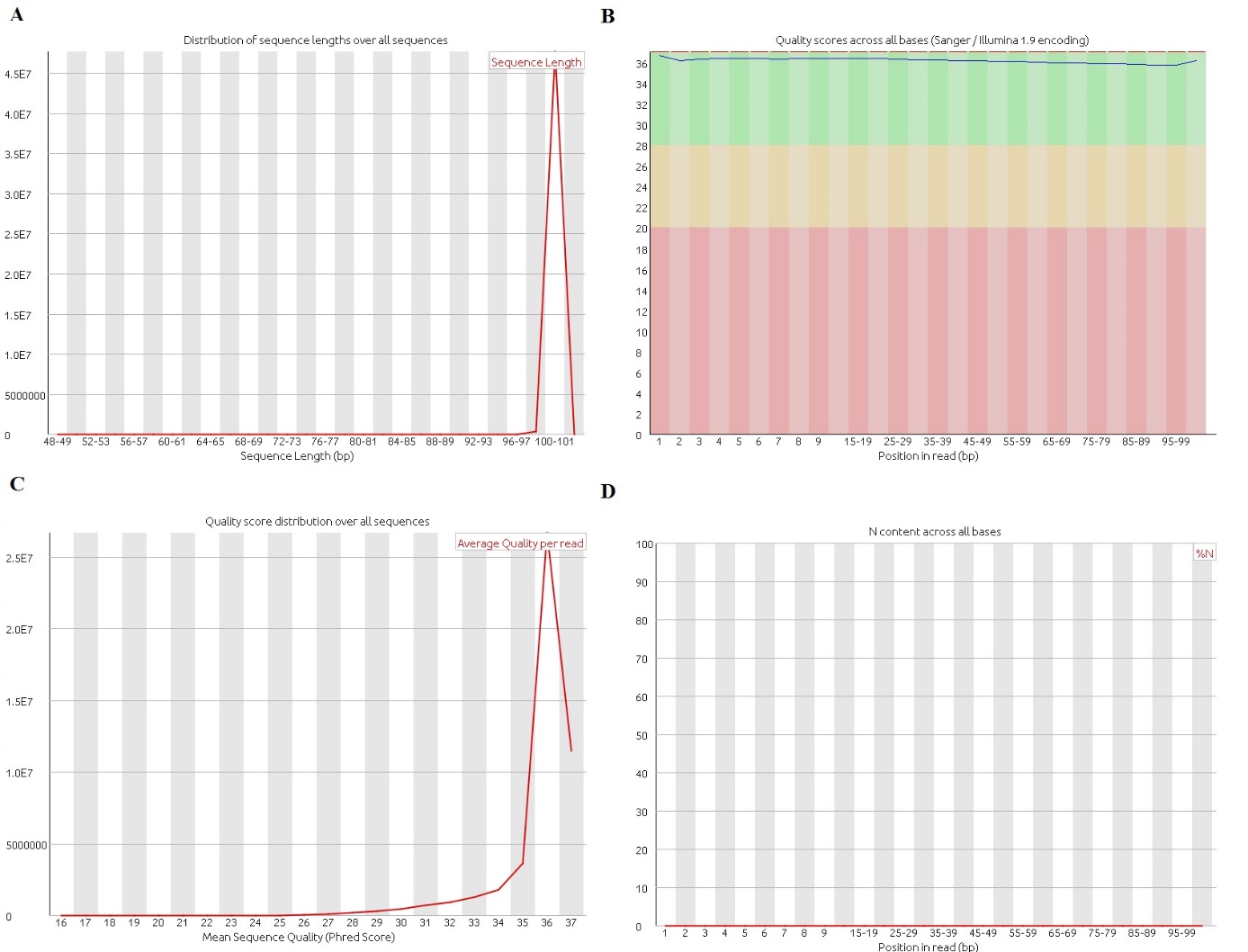


**Figure F6. Sequencing quality report of RNA-seq of *Androctonus Crassicauda* datasets by Fastqc.** A) Sequence length distribution of raw reads from mature female *A. Crassicauda.* B) Per base quality of raw reads from mature female *A. Crassicauda.* C) Per sequence quality of raw reads from mature female *A. Crassicauda.* D) Per base n content of raw reads from mature female *A. Crassicauda.*


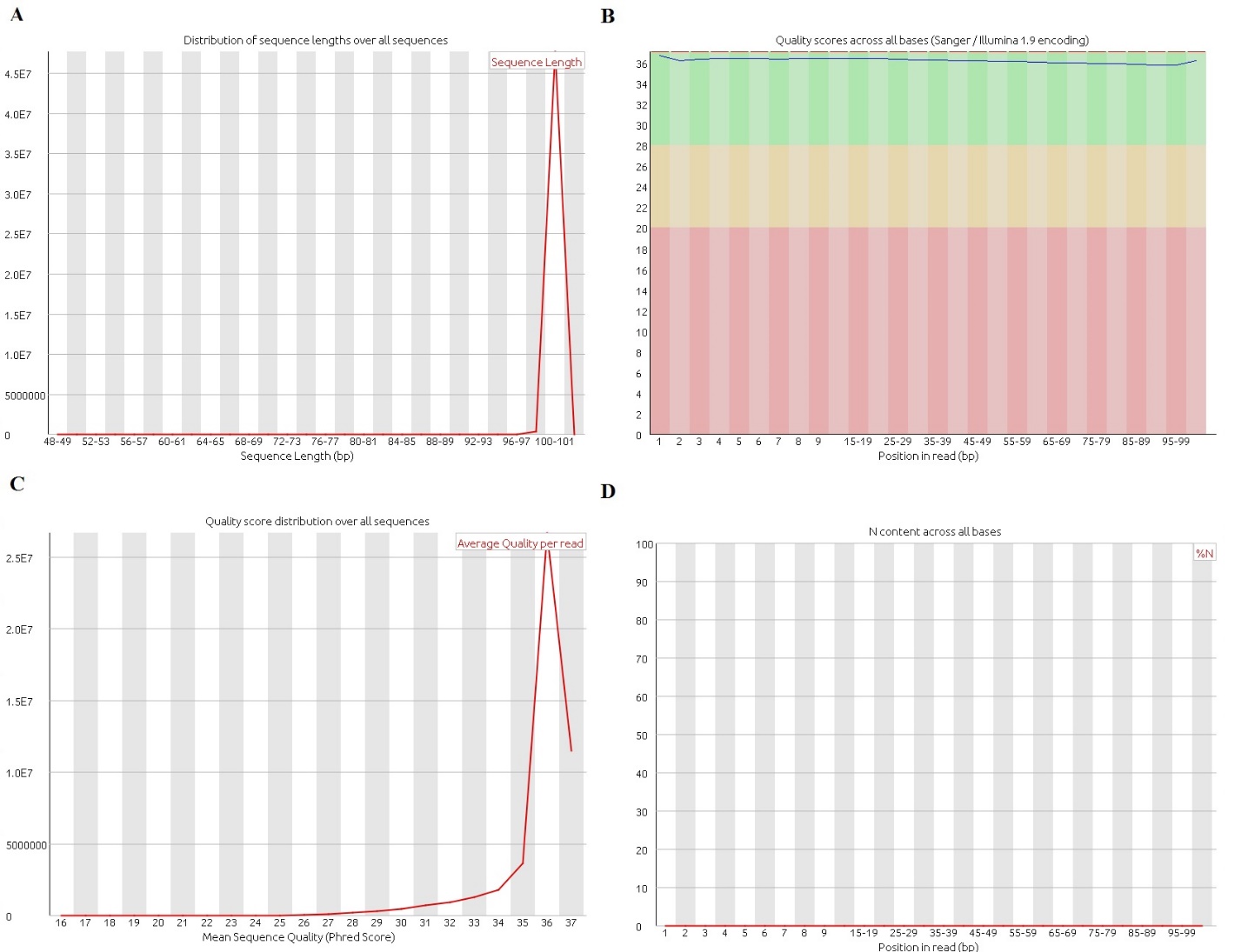

Supplement: Supplementary file 5 — Supplementary Information 5. [file 41598_2021_83815_MOESM5_ESM.docx]
